# Supplementary material for: A cowpea mosaic virus adjuvant conjugated to liposomes loaded with tumor cell lysates as an ovarian cancer vaccine
Source: Nat Commun. 2025 May 30;16:5047. doi: 10.1038/s41467-025-60239-w (PMC12125389; doi:10.1038/s41467-025-60239-w)
Supplement: Supplementary file 1 — Supplementary Information [file 41467_2025_60239_MOESM1_ESM.pdf]

Supplementary information for manuscript

A cowpea mosaic virus adjuvant conjugated to liposomes loaded with tumor cell lysates as an ovarian cancer vaccine

Authors:

Zhongchao Zhao[1,2,3], Debbie Ledezma[1,2,3], Jessica Fernanda Affonso de Oliveira[1,2,3], Anthony O. Omole[1,2,3], Nicole F. Steinmetz[1,2,3,4,5,6,7,8#]

[1]Department of NanoEngineering, University of California, San Diego, 9500 Gilman Dr., La Jolla, CA, 92093 USA

[2]Center for Nano-ImmunoEngineering, University of California, San Diego, 9500 Gilman Dr., La Jolla, CA, 92093 USA

[3]Moores Cancer Center, University of California, San Diego, 9500 Gilman Dr., La Jolla, CA, 92093 USA

[4]Department of Bioengineering, University of California, San Diego, 9500 Gilman Dr., La Jolla, CA, 92093 USA

[5]Department of Radiology, University of California, San Diego, 9500 Gilman Dr., La Jolla, CA, 92093 USA

[6]Institute for Materials Discovery and Design, University of California, San Diego, 9500 Gilman Dr., La Jolla, CA, 92093 USA

[7]Center for Engineering in Cancer, University of California, San Diego, 9500 Gilman Dr., La Jolla, CA, 92093 USA

[8]Shu and K.C. Chien and Peter Farrell Collaboratory, University of California, San Diego, 9500 Gilman Dr., La Jolla, CA, 92093 USA

[#] Corresponding author: nsteinmetz@ucsd.edu

ORCID: https://orcid.org/0000-0002-0130-0481

Supplementary methods

Production of Oxidized tumor cell lysates and irradiated tumor cell lysates

ID8-Defb29/Vegf-a-Luc cells were cultured in RPMI 1640 medium with L-glutamine supplemented with 10% (v/v) FBS, 1% (v/v) Pen/Strep, 1 mM sodium pyruvate, and 0.05 mM β-mercaptoethanol in an incubator at 37 °C with a 5% CO_2 atmosphere.

Oxidized tumor cell lysates

ID8-Defb29/Vegf-a-Luc cells were harvested in trypsin-EDTA, washed 3 times using PBS, resuspended in growth media containing 60 μM HOCl (ThermoFisher Scientific) for 1 h at 37 °C[1]. Cells were then washed 3 times using PBS and adjusted to 1 × 10^7 cells/mL in 50 mM HEPES (pH 7.4). After five freeze–thaw cycles using liquid nitrogen and a 37 °C water bath, cell debris was removed by two rounds of centrifugation (14,000 × g, 10 min, room temperature). Oxidized TCL was recovered and stored at -80 °C.

Irradiated tumor cell lysates

ID8-Defb29/Vegf-a-Luc cells were harvested in trypsin-EDTA, washed 3 times using PBS, resuspended in growth media, and irradiated at 70 Gray using a Cs-137 X-ray source (10 Gy per 1.18 min for 8.26 min). Cells were further cultured for 12 hours, then harvested and washed 3 times using PBS. Cells at 1 × 10^7 cells/mL in 50 mM HEPES pH 7.4 then underwent five freeze–thaw cycles using liquid nitrogen and a 37 °C water bath. Cell debris was removed by two rounds of centrifugation (14,000 × g, 10 min, room temperature). Irradiated TCL was recovered and stored at -80 °C.

Supplementary Figures

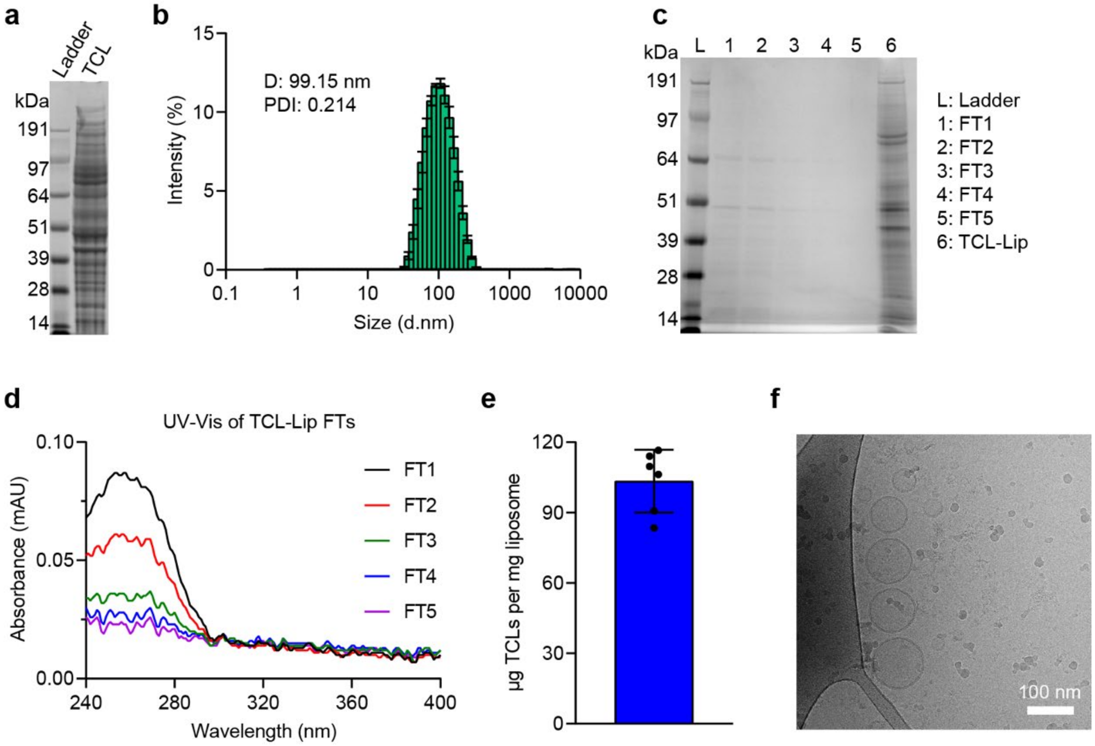

Supplementary Fig. 1. Characterization of TCL and TCL-Lip. (a) NuPAGE of TCL isolated from ID8-Defb29/Vegf-a-Luc murine ovarian cancer cells. (b) DLS of 100 nm TCL-Lip; n = 3 repeated measurements of the same sample, data are expressed as mean +/- SD. (c) NuPAGE analysis of flow-through fractions (FT1–5) from five cycles of TFF and purified TCL-Lip. (d) UV-Vis of FT fractions from TFF. (e) Quantification of the amount of loaded TCL per mg liposomes; n = 6 independent experiments, data are expressed as mean +/- SD. (f) Cryo-EM imaging of TCL-Lip. Three independent experiments were performed with similar results (a-d, f). Source data are provided as a Source Data file (a,c,e).

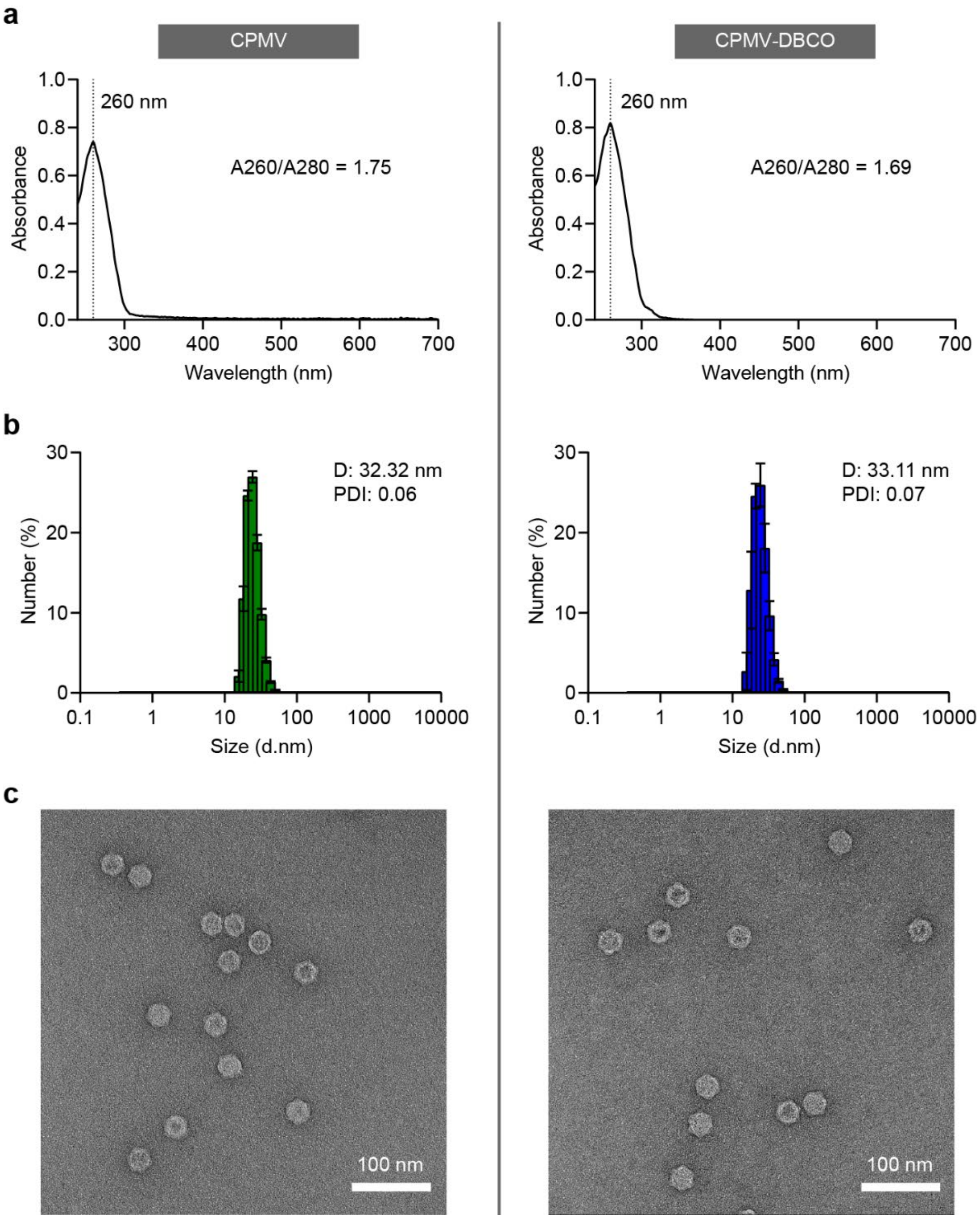

Supplementary Fig. 2. Characterization of CPMV and CPMV-DBCO. (a) UV-Vis of CPMV and CPMV-DBCO. (b) DLS of CPMV and CPMV-DBCO; n = 3 repeated measurements of the same sample, data are expressed as mean +/- SD. (c) TEM of CPMV and CPMV-DBCO. Three independent experiments were performed with similar results (a-c).

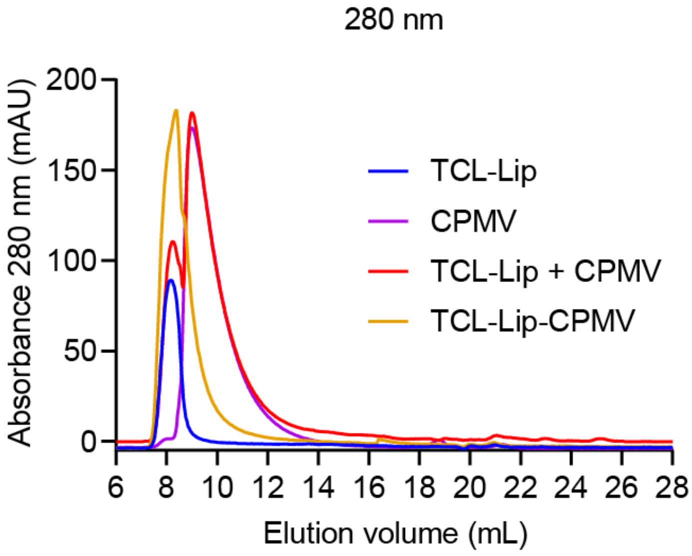

Supplementary Fig. 3. Elution profiles of the SEC analysis of TCL-Lip, CPMV, TCL-Lip + CPMV, and TCL-Lip-CPMV as shown in Fig. 1d. Three independent experiments were performed with similar results.

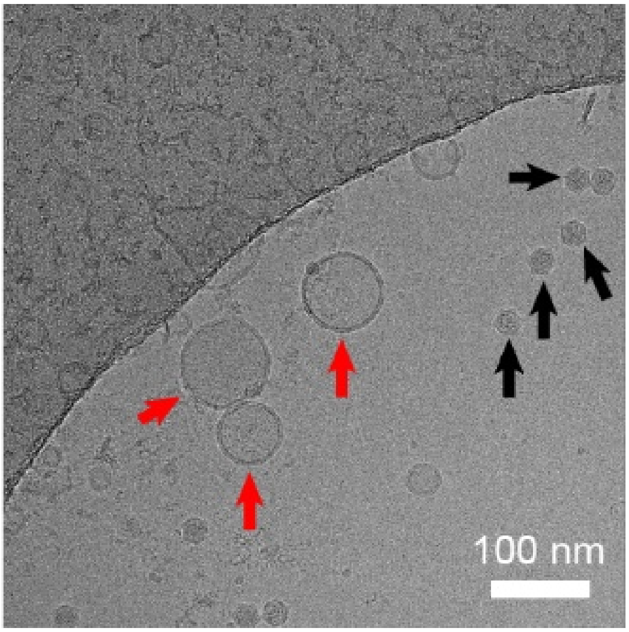

Supplementary Fig. 4. Cryo-EM of TCL-Lip + CPMV mixture. TCL-loaded liposomes (TCL-Lip) are indicated by red arrows and CPMV particles are indicated by black arrows. Three independent experiments were performed with similar results.

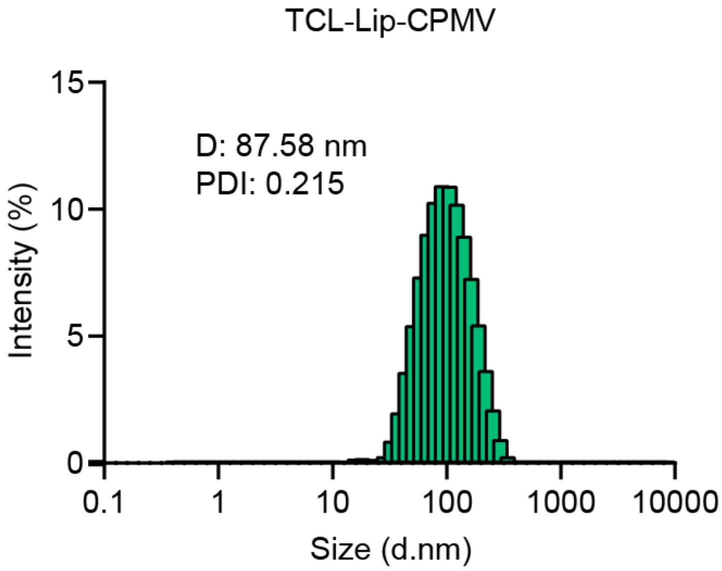

Supplementary Fig. 5. DLS of CPMV-Lip-CPMV; n = 3 repeated measurements of the same sample, data are expressed as mean +/- SD.

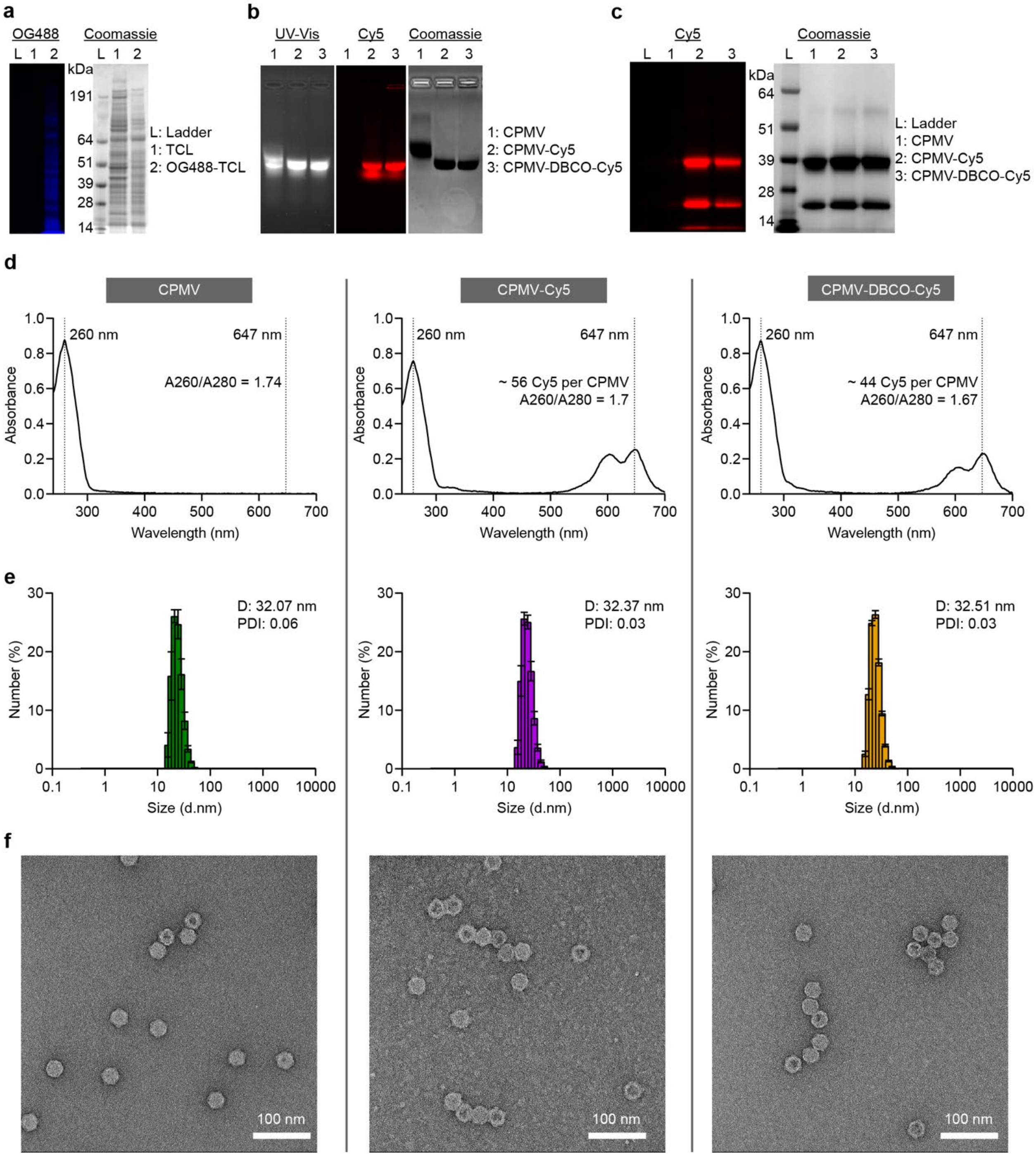

Supplementary Fig. 6. Characterization of labeled CPMV formulations. (a) NuPAGE analysis of TCL and OG488-labelled TCL. Agarose gel electrophoresis (b) and NuPAGE (c) analysis of CPMV, CPMV-Cy5, and CPMV-DBCO-Cy5. (d) UV-Vis analysis of CPMV, CPMV-Cy5, and CPMV-DBCO-Cy5. (d) DLS analysis of CPMV, CPMV-Cy5, and CPMV-DBCO-Cy5; n = 3 repeated measurements of the same sample, data are expressed as mean +/- SD. (e) TEM images of CPMV, CPMV-Cy5, and CPMV-DBCO-Cy5. Three independent experiments were performed with similar results. Source data are provided as a Source Data file (a-c).

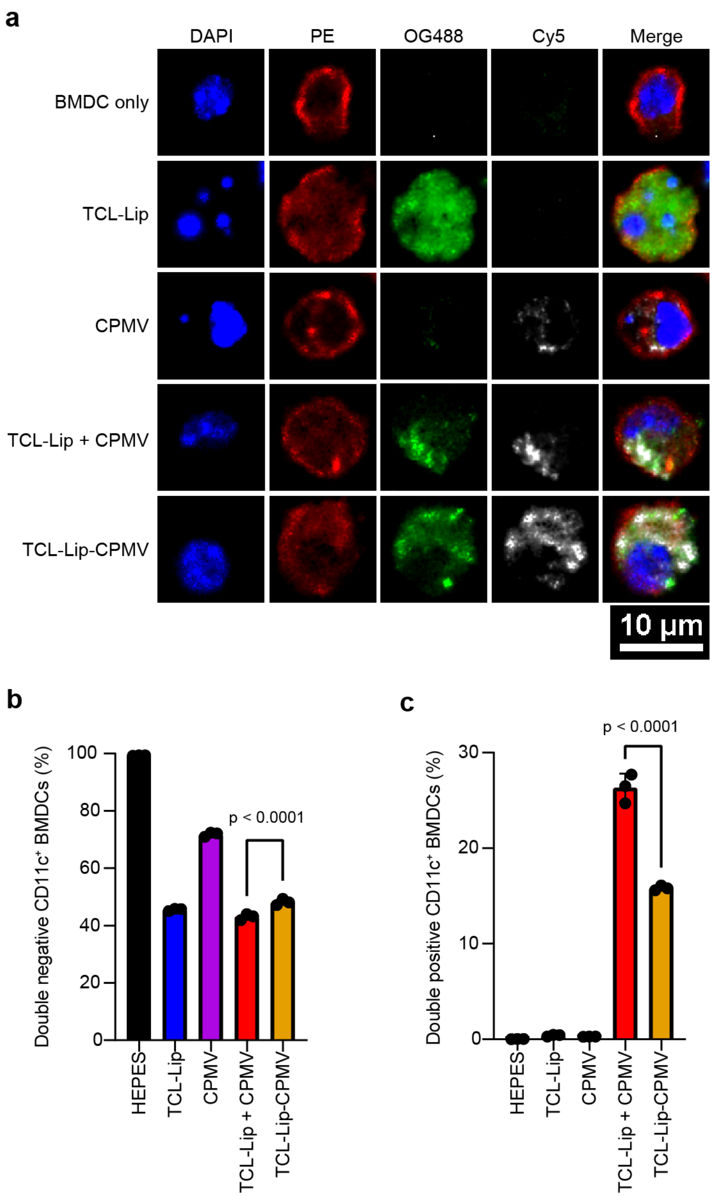

Supplementary Fig. 7. Co-delivery of OG488-TCL-Lip and CPMV-Cy5 to BMDCs. (a) Confocal micrograph images of BMDCs after incubation for 1 h with OG488-TCL-Lip, CPMV-Cy5, OG488-TCL-Lip + CPMV-Cy5, and OG488-TCL-Lip-CPMV-Cy5, showing the co-delivery of OG488-TCL-Lip and CPMV-Cy5. Flow cytometry analysis of double-negative BMDCs, i.e. negative for TCL-Lip and CPMV (b) and double-positive BMDCs, i.e. positive for TCL and CPMV (c) after incubation for 1 h with OG488-TCL-Lip, CPMV-Cy5, OG488-TCL-Lip + CPMV-Cy5, and OG488-TCL-Lip-CPMV-Cy5; n = 3 independent experiments, data are expressed as mean +/- SD. The results at 24 h post incubation were shown in Fig. 2c-e. Three independent experiments were performed with similar results (a). Statistical significance was determined by ordinary one-way ANOVA. Source data are provided as a Source Data file (b,c).

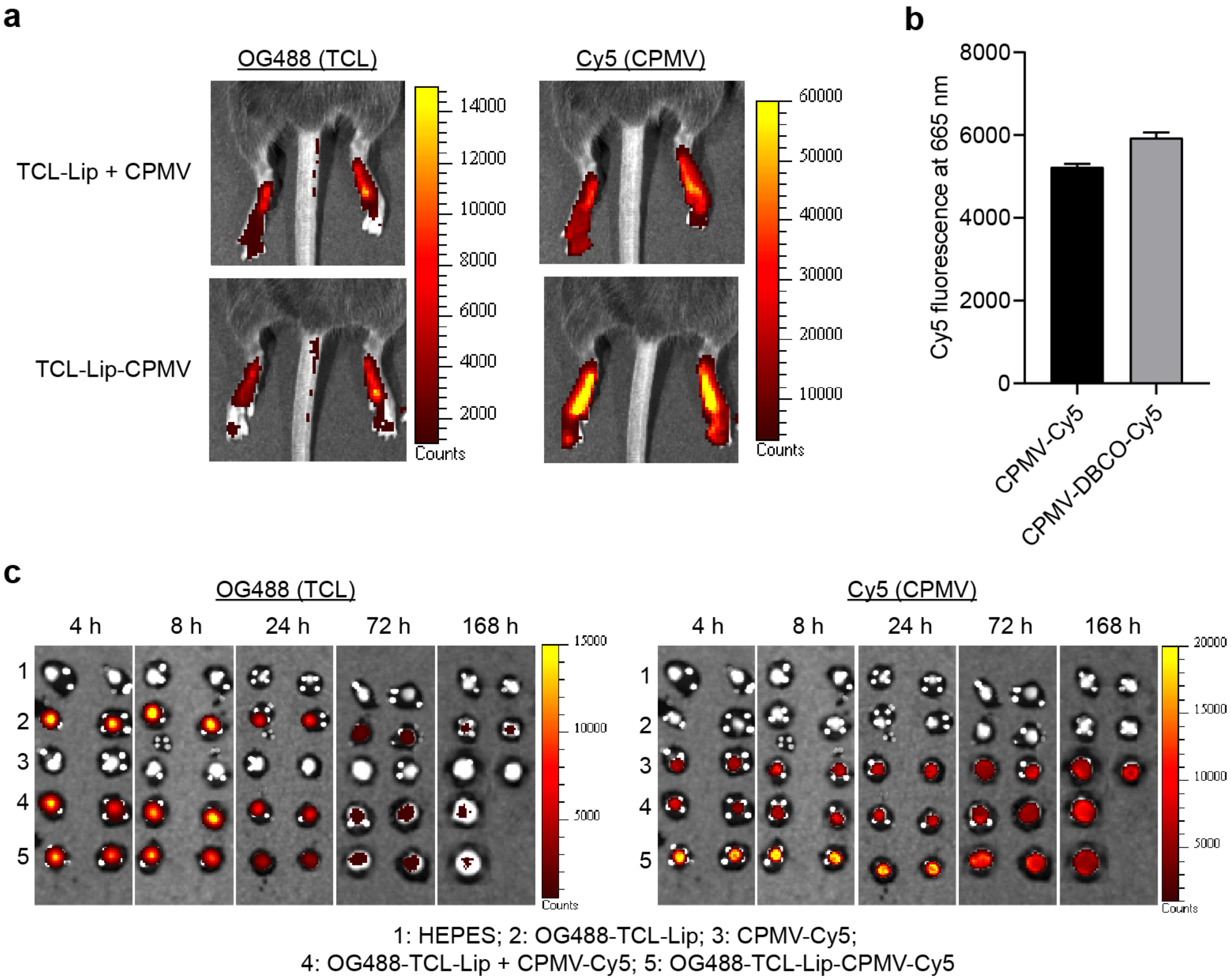

Supplementary Fig. 8. Lymph node homing of TCL-Lip-CPMV formulations. (a) Representative IVIS images of footpads following the injection of labeled TCL-Lip + CPMV and TCL-Lip-CPMV. (b) Quantification of Cy5 fluorescence intensity (1 mg/mL of CPMV-Cy5 and CPMV-DBCO-Cy5) showing the formulations have comparable fluorescence intensity; n = 3 independent experiments, data are expressed as mean +/- SD. (c) IVIS images of harvested popliteal draining lymph nodes following footpad injections of OG488-TCL-Lip, CPMV-Cy5, OG488-TCL-Lip + CPMV-Cy5, and OG488-TCL-Lip-CPMV-Cy5. OG488-TCL was imaged using the GFP filter and CPMV-Cy5 was imaged using the Cy5.5 filter. Source data are provided as a Source Data file (b).

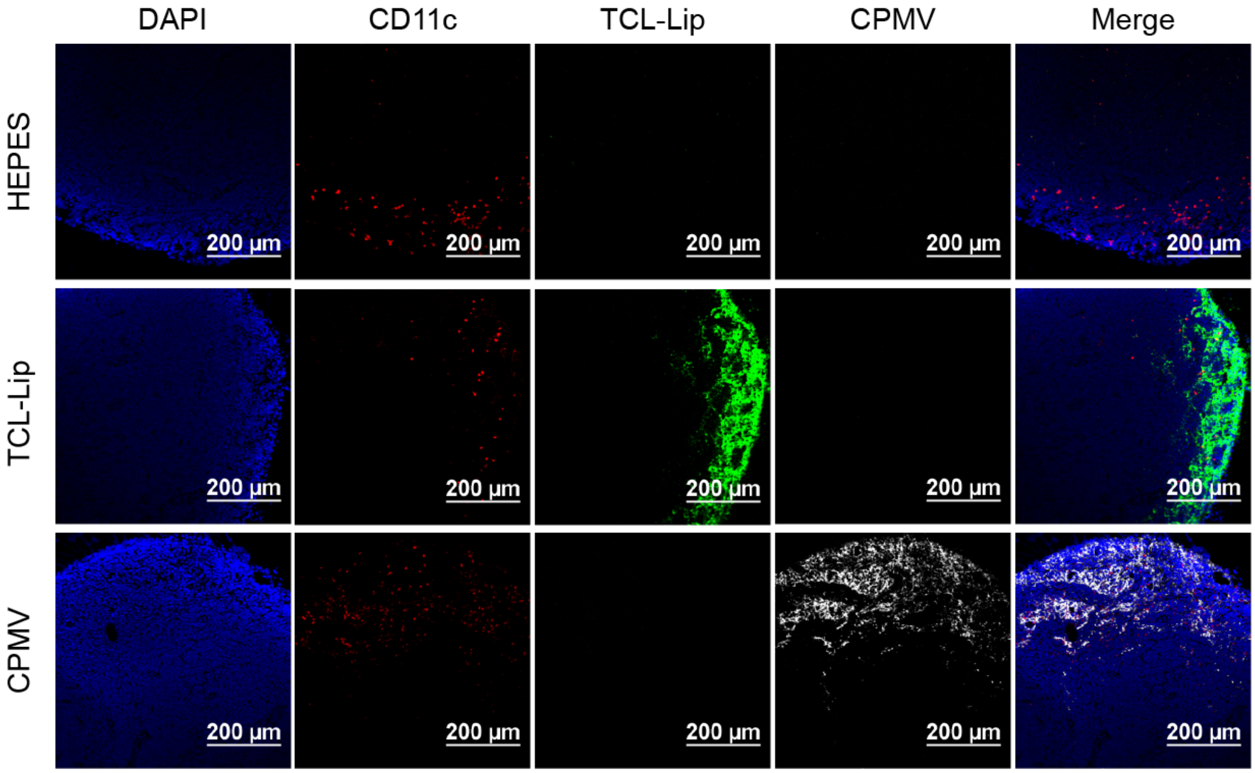

Supplementary Fig. 9. Immunofluorescence images of harvested lymph node cryo-sections: HEPES, TCL-Lip and CPMV groups 4 h after footpad injections. TCL-Lip + CPMV and TCL-Lip-CPMV groups are presented in Fig. 3a. Dendritic cells are stained using a PE-conjugated anti-CD11c antibody. CPMV and TCL-Lip are visualized using the OG488 and Cy5 label, respectively.

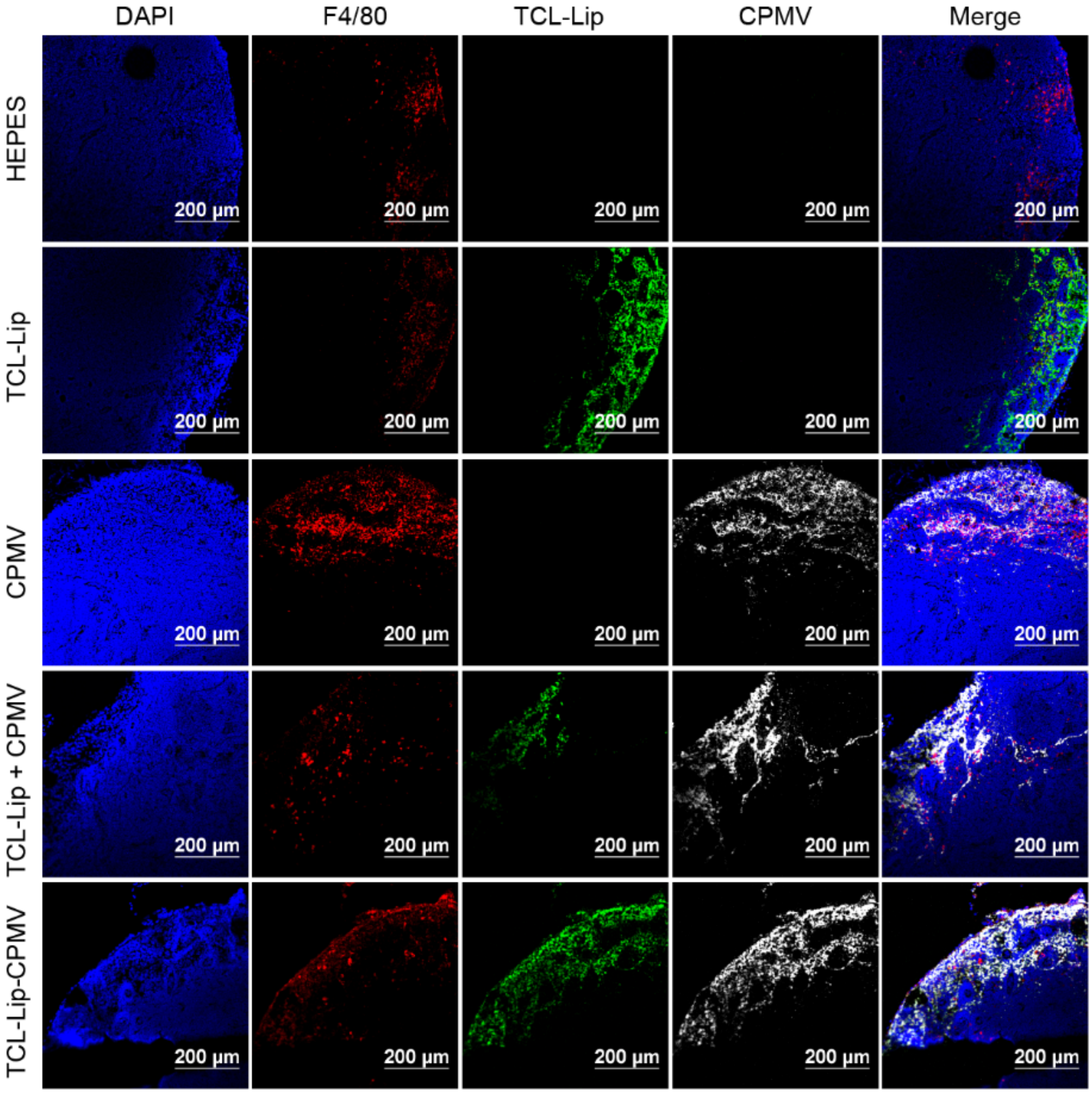

Supplementary Fig. 10. Immunofluorescence images of harvested lymph node cryo-sections 4 h after footpad injections. Macrophages are stained using a PE-conjugated anti-F4/80 antibody. CPMV and TCL-Lip are visualized using the OG488 and Cy5 label, respectively.

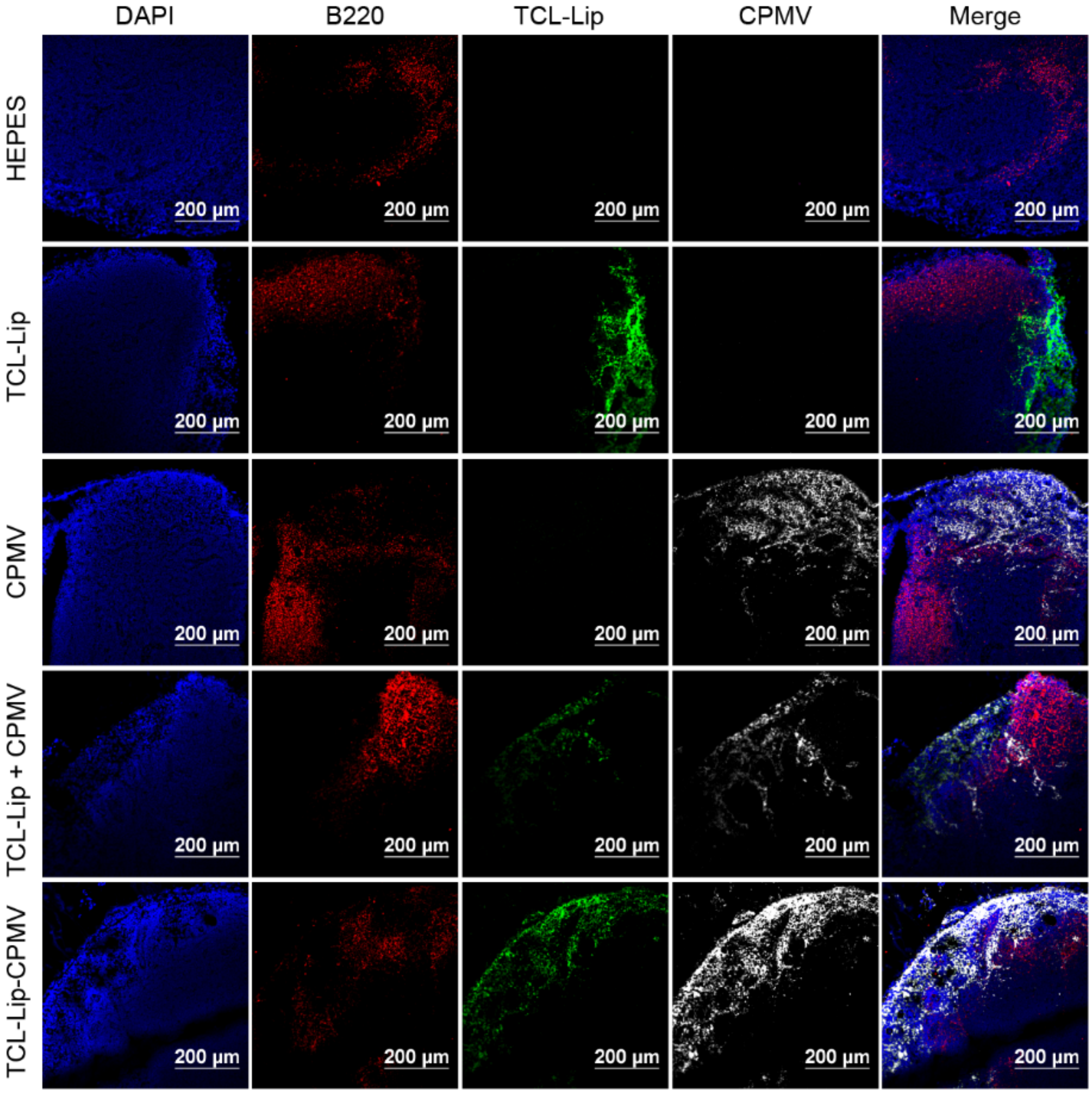

Supplementary Fig. 11. Immunofluorescence images of harvested lymph node cryo-sections 4 h after footpad injections. B cells are stained using a PE-conjugated anti-B220 antibody. CPMV and TCL-Lip are visualized using the OG488 and Cy5 label, respectively.

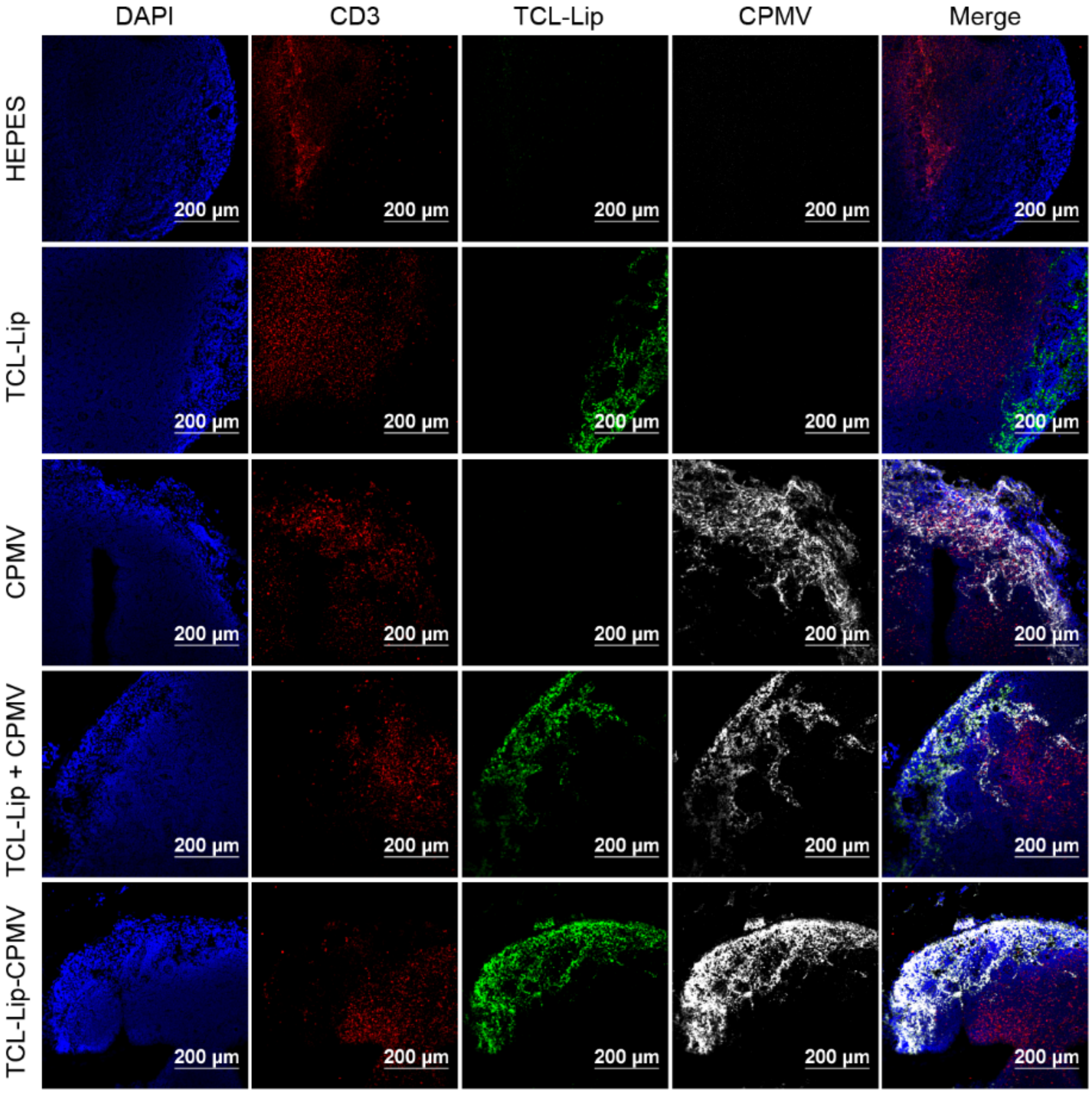

Supplementary Fig. 12. Immunofluorescence images of harvested lymph node cryo-sections 4 h after footpad injections. T cells are stained using a PE-conjugated anti-CD3 antibody. CPMV and TCL-Lip are visualized using the OG488 and Cy5 label, respectively.

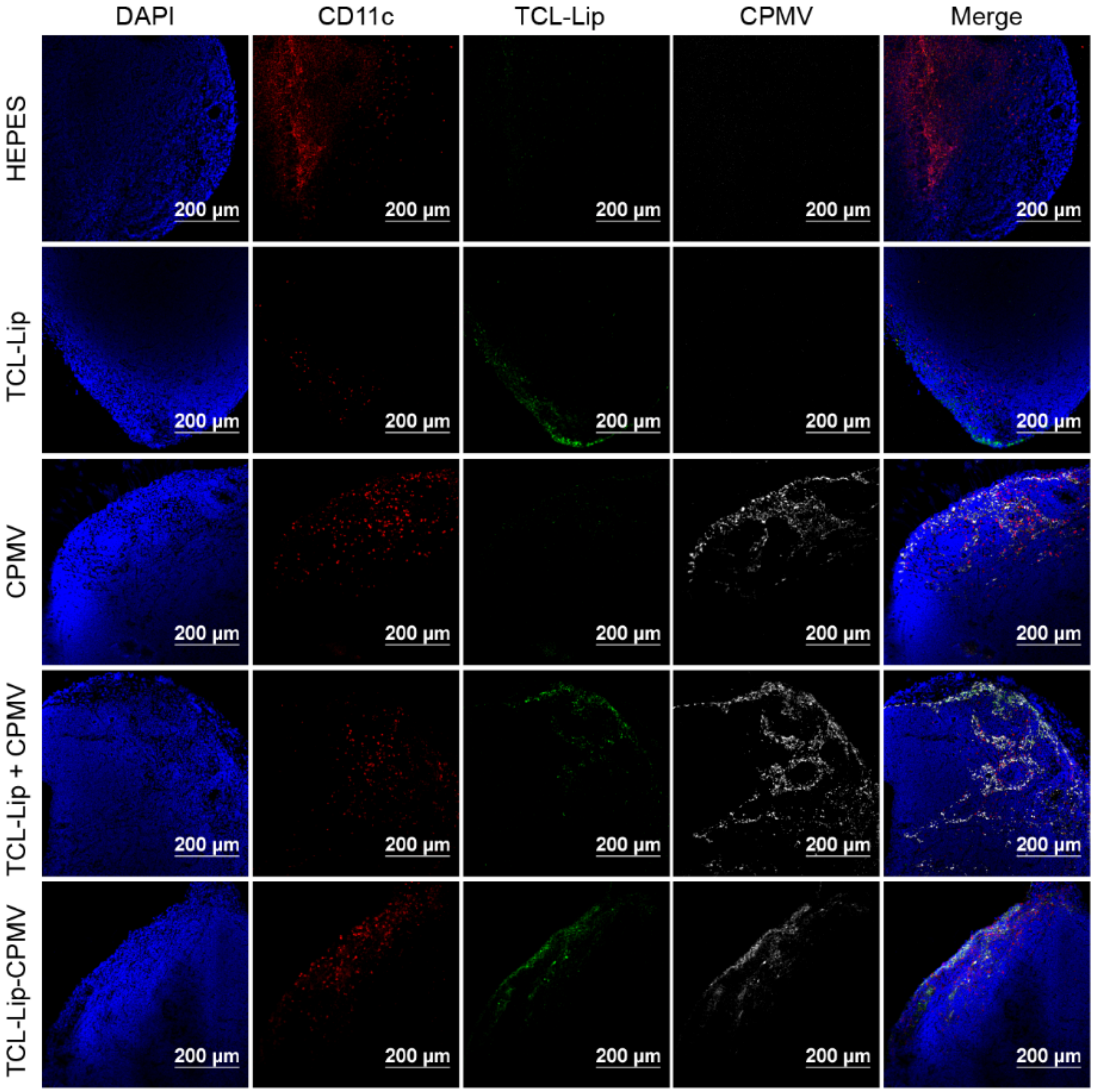

Supplementary Fig. 13. Immunofluorescence images of harvested lymph node cryo-sections 24 h after footpad injections. Dendritic cells are stained using a PE-conjugated anti-CD11c antibody. CPMV and TCL-Lip are visualized using the OG488 and Cy5 label, respectively.

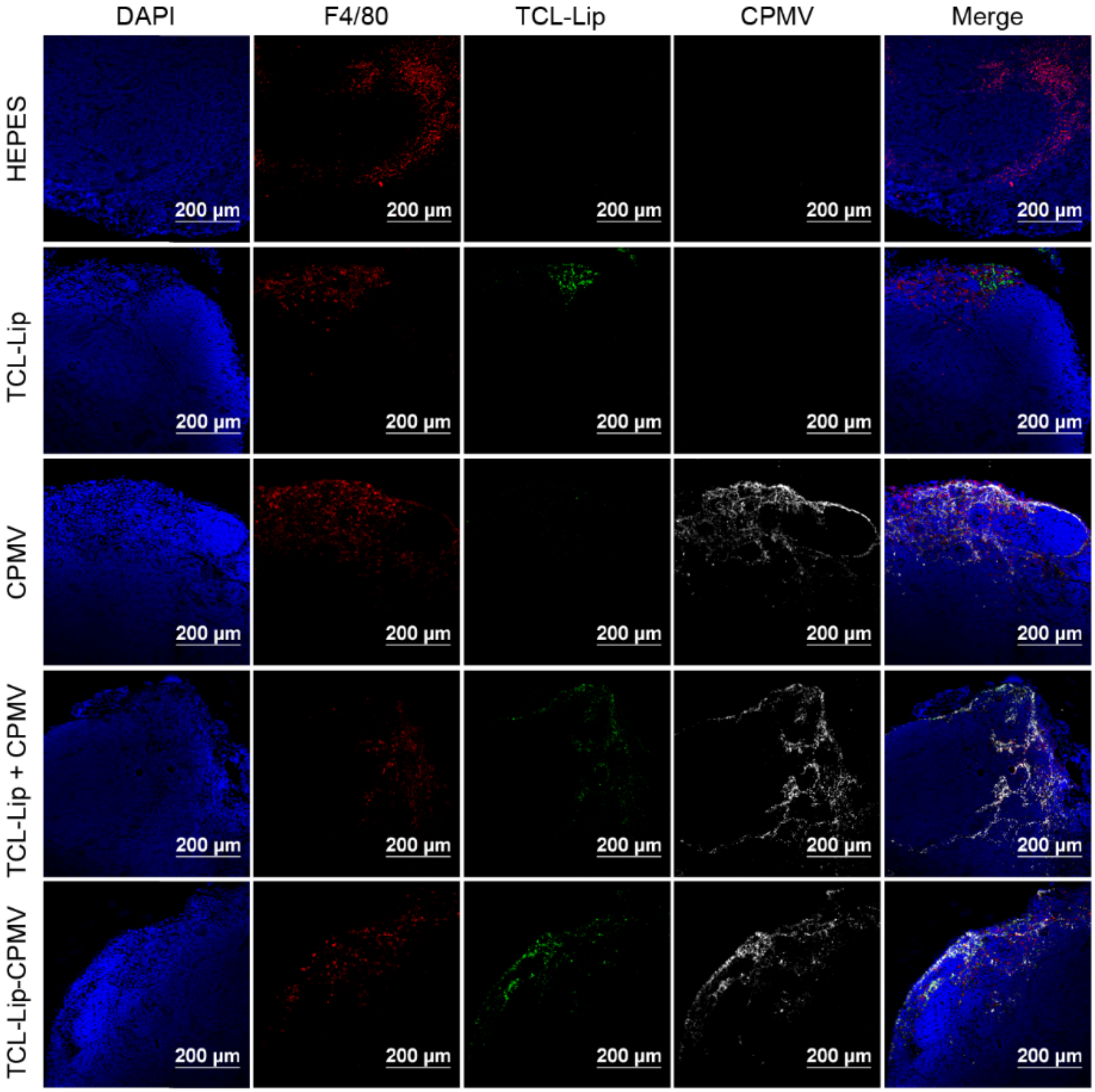

Supplementary Fig. 14. Immunofluorescence images of harvested lymph node cryo-sections 24 h after footpad injections. Macrophages are stained using a PE-conjugated anti-F4/80 antibody. CPMV and TCL-Lip are visualized using the OG488 and Cy5 label, respectively.

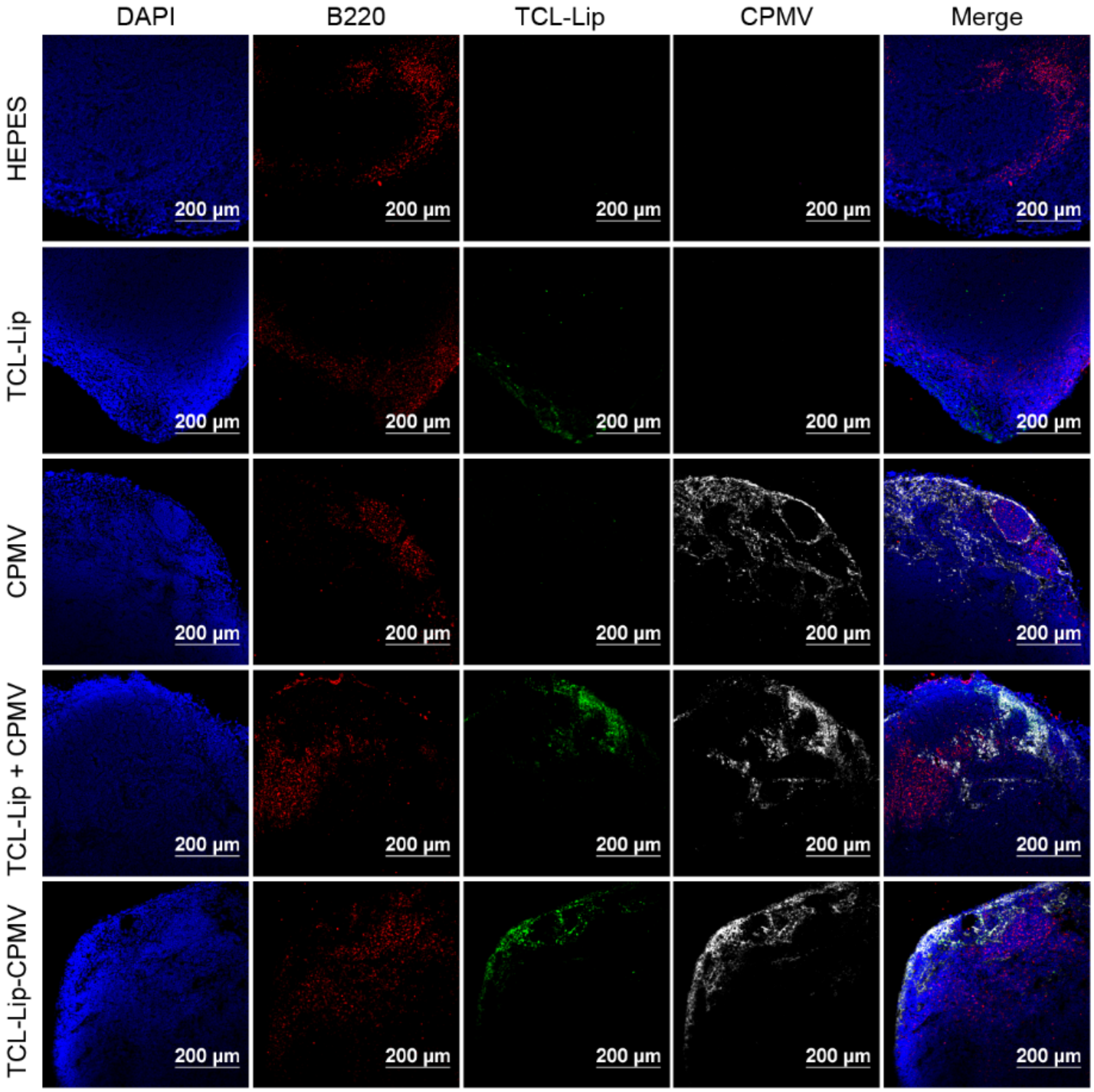

Supplementary Fig. 15. Immunofluorescence images of harvested lymph node cryo-sections 24 h after footpad injections. B cells are stained using a PE-conjugated anti-B220 antibody. CPMV and TCL-Lip are visualized using the OG488 and Cy5 label, respectively.

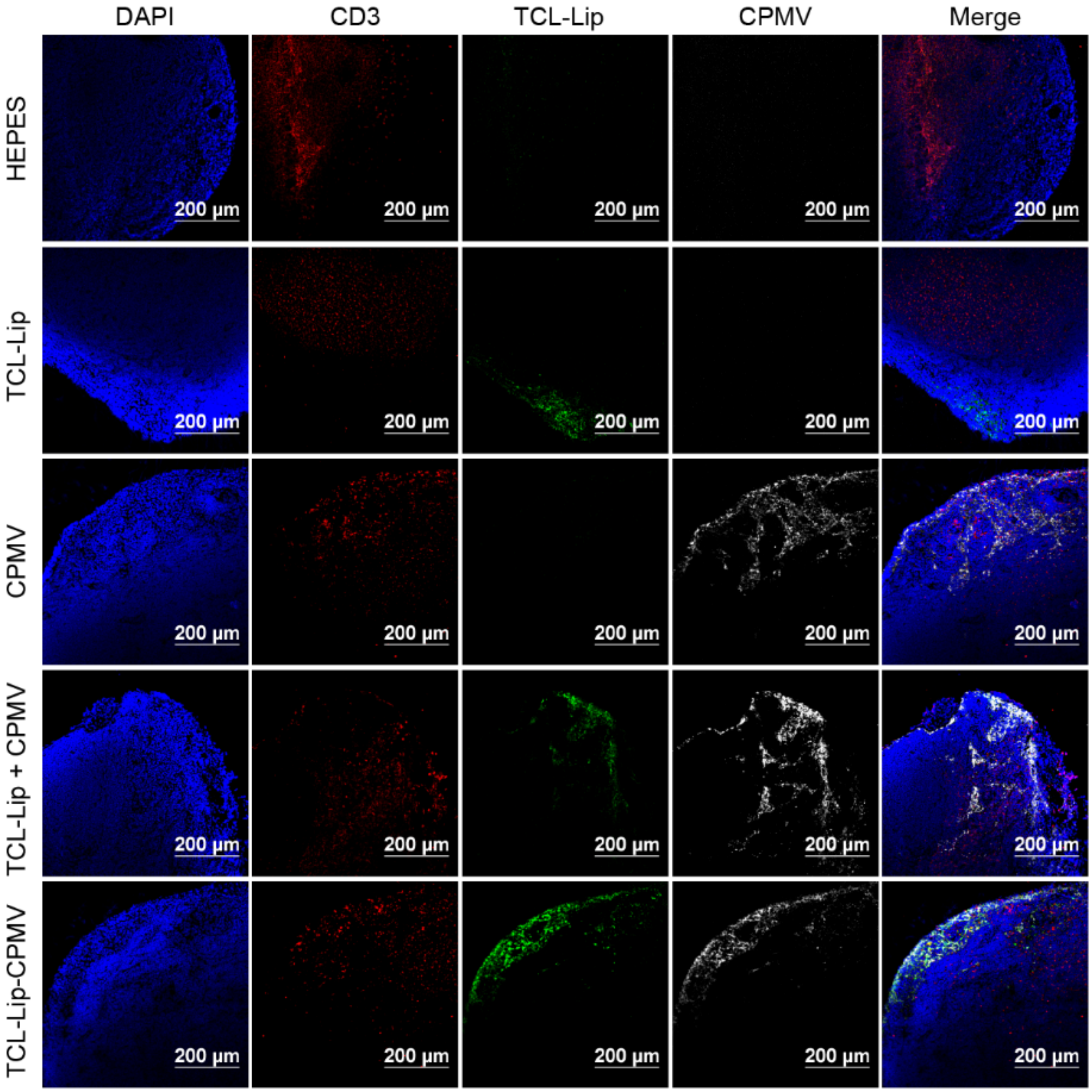

Supplementary Fig. 16. Immunofluorescence images of harvested lymph node cryo-sections 24 h after footpad injections. T cells are stained using a PE-conjugated anti-CD3 antibody. CPMV and TCL-Lip are visualized using the OG488 and Cy5 label, respectively.

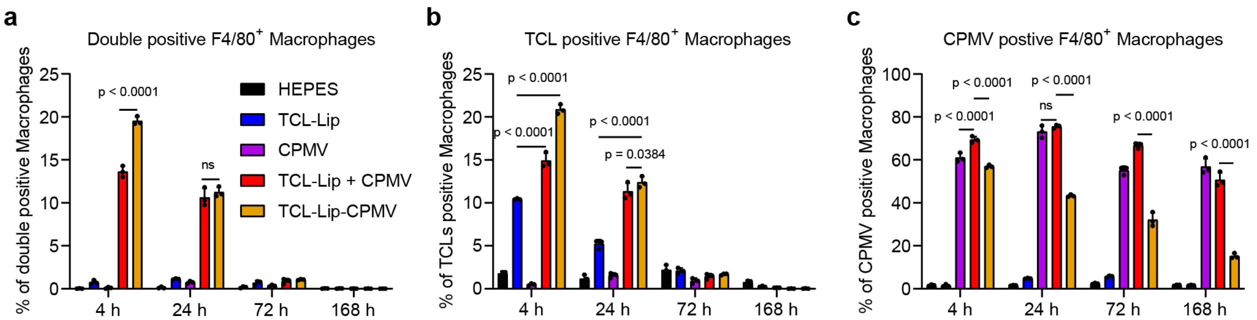

Supplementary Fig. 17. Flow cytometry analysis of F4/80⁺ macrophages within harvested lymph nodes: (a) double-positive for OG488-TCL and CPMV-Cy5, (b) positive for OG488-TCL, and (c) positive for CPMV-Cy5; n = 3 independent experiments, data are expressed as mean +/- SD. Statistical significance was determined by ordinary one-way ANOVA. Source data are provided as a Source Data file.

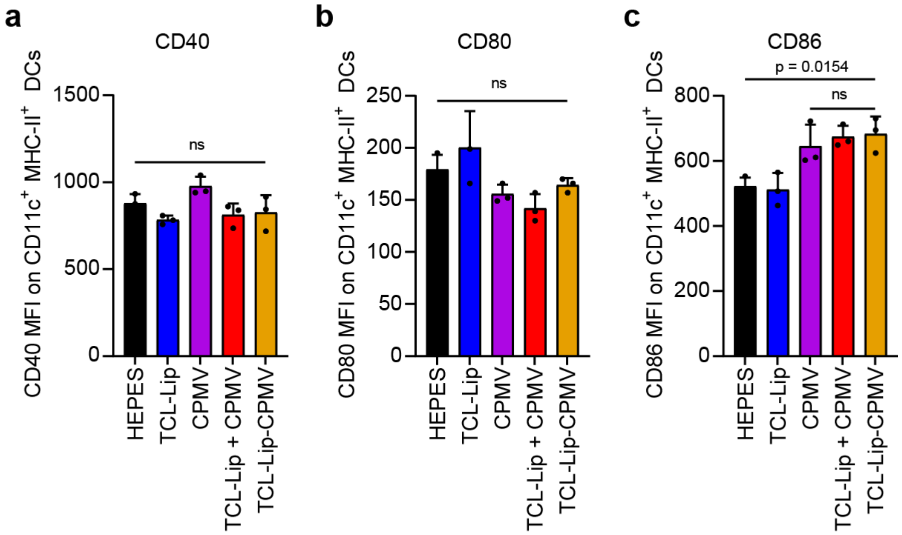

Supplementary Fig. 18. Activation of dendritic cells (DCs) within popliteal draining lymph nodes 4 h after footpad injections. The activation status of CD11c$^+$ MHC-II$^+$DCs was measured by flow cytometry based on the expression levels of (a) CD40, (b) CD80, and (c) CD86 surface markers; n = 3 independent experiments, data are expressed as mean +/- SD. Statistical significance was determined by ordinary one-way ANOVA. Source data are provided as a Source Data file.

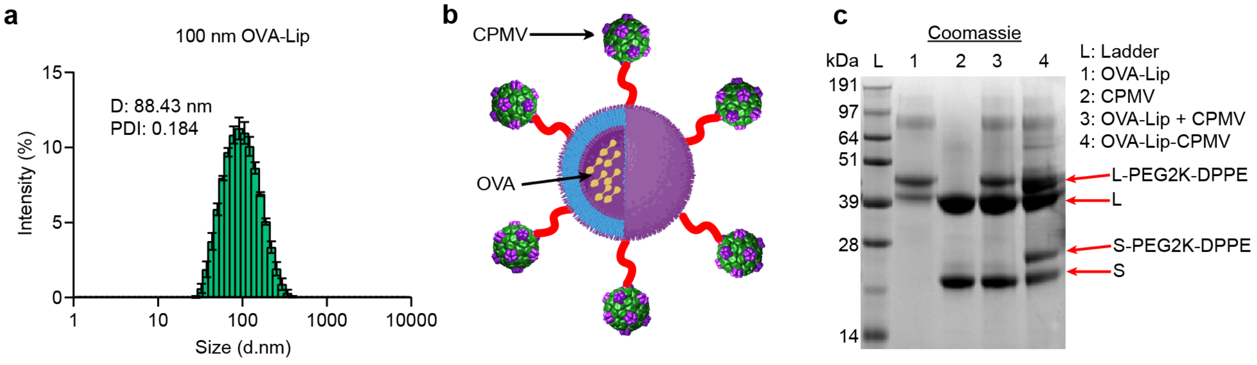

Supplementary Fig. 19. Characterization of formulations containing ovalbumin (OVA). (a) DLS analysis of 100-nm OVA-Lip; n = 3 repeated measurements of the same sample, data are expressed as mean +/- SD. (b) A representation of the OVA-Lip-CPMV formulation. Figure is generated using Biorender and Adobe Illustrator. (c) NuPAGE analysis of OVA-Lip-CPMV to confirm the conjugation of CPMV to OVA-Lip as evident by the L-PEG2K-DPPE and S-PEG2K-DPPE bands. Three independent experiments were performed with similar results (a,c). Part of (b) is generated using BioRender. Source data are provided as a Source Data file (c).

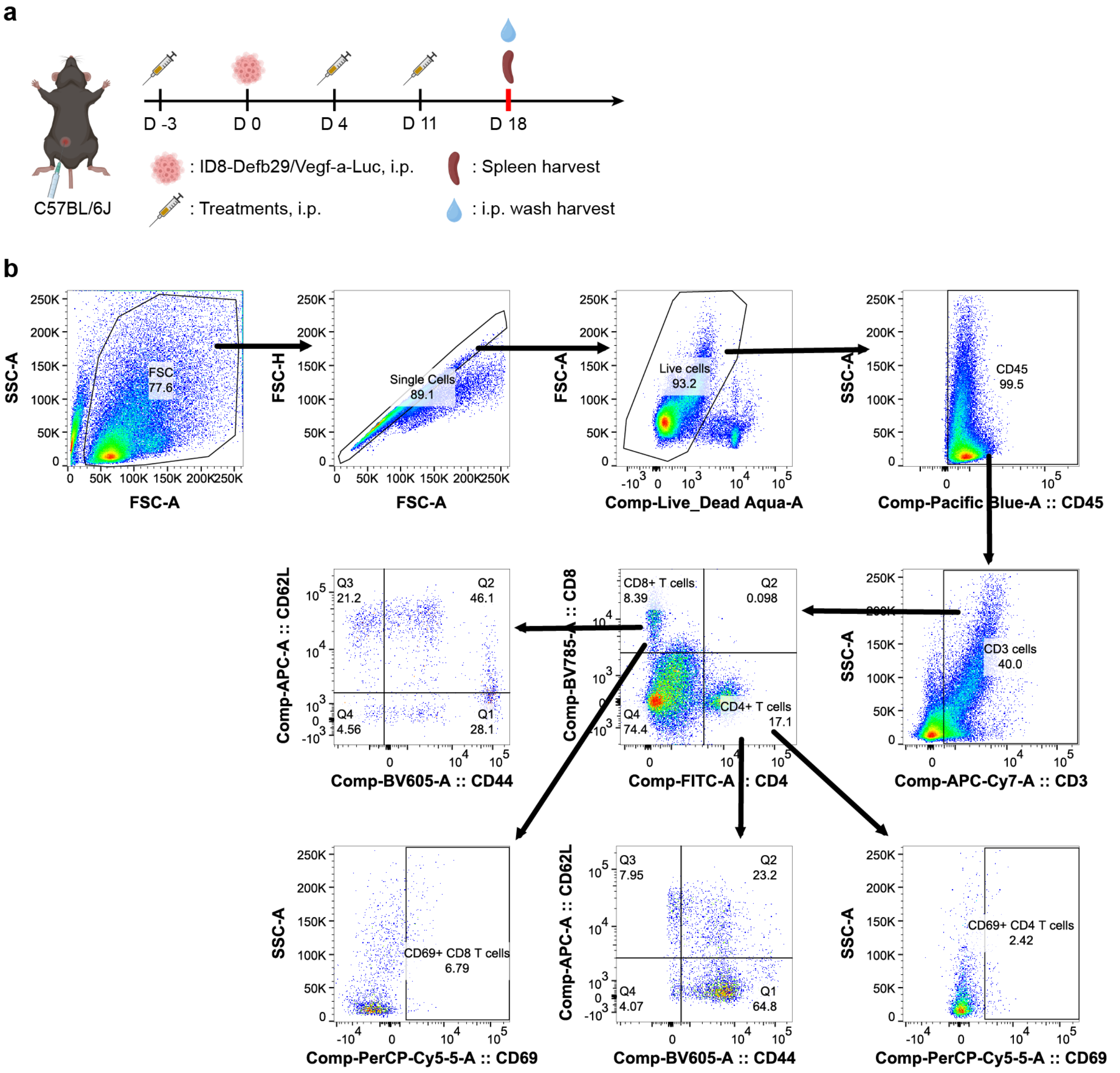

Supplementary Fig. 20. Analysis of CD4$^+$ and CD8$^+$ T cells. (a) Schedules for treatments, tumor inoculations, i.p. wash and spleen harvest. (b) Gating strategies for CD4$^+$ and CD8$^+$ T cells and their subsets. Part of (a) is generated using BioRender.

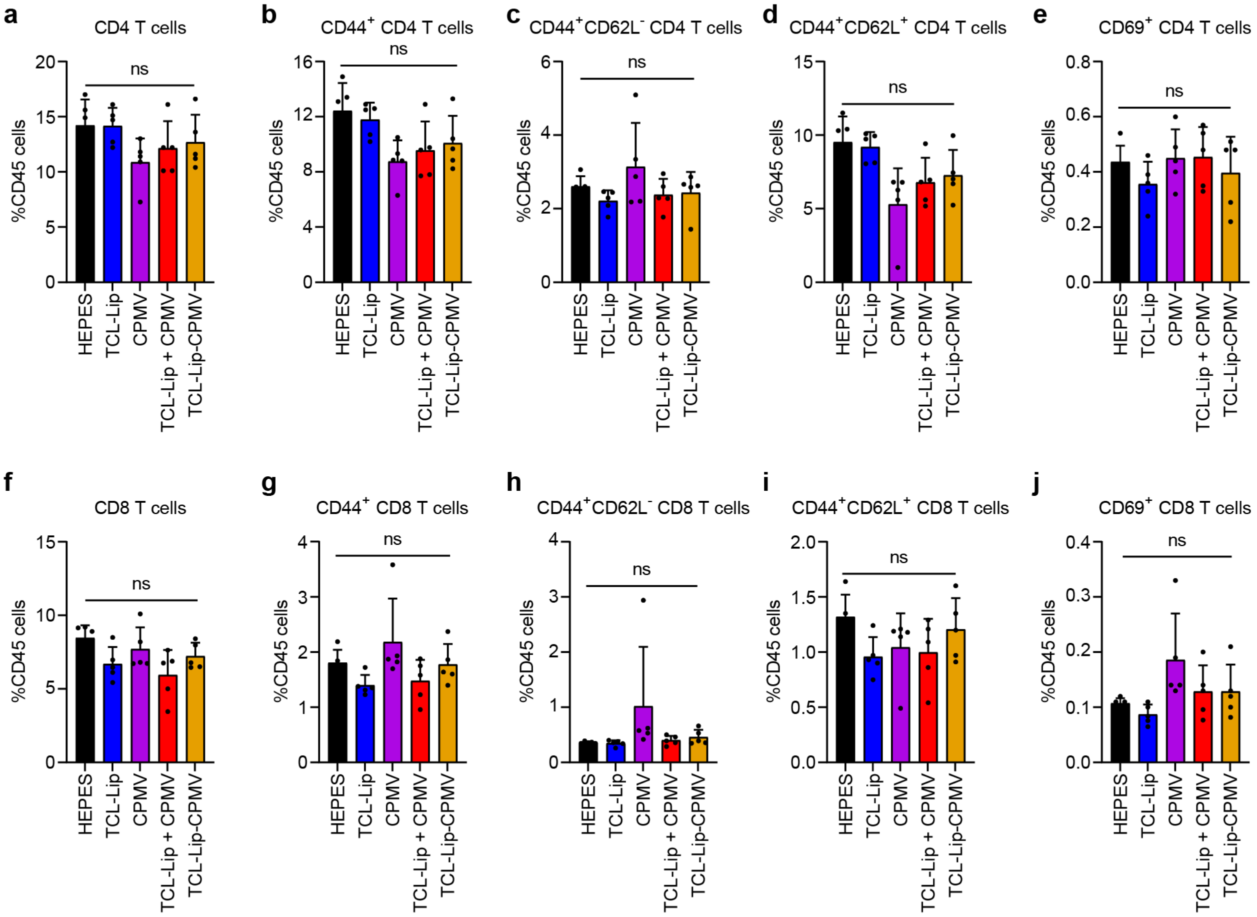

Supplementary Fig. 21. CD4$^+$ and CD8$^+$ T cells in spleens after treatment; n = 5 independent experiments. (a) Percentage of CD3$^+$CD4$^+$ T cells among CD45$^+$ immune cells. (b) Percentage of CD44$^+$ memory CD4$^+$ T cells among CD45$^+$ immune cells. (c) Percentage of CD44$^+$CD62L$^-$ effector memory CD4$^+$ T cells among CD45$^+$ immune cells. (d) Percentage of CD44$^+$CD62L$^+$ central memory CD4$^+$ T cells among CD45$^+$ immune cells. (e) Percentage of CD69$^+$ activated CD4$^+$ T cells among CD45$^+$ immune cells. (f) Percentage of CD3$^+$CD8$^+$ T cells among CD45$^+$ immune cells. (g) Percentage of CD44$^+$ memory CD8$^+$ T cells among CD45$^+$ immune cells. (h) Percentage of CD44$^+$CD62L$^-$ effector memory CD8$^+$ T cells among CD45$^+$ immune cells. (i) Percentage of CD44$^+$CD62L$^+$ central memory CD8$^+$ T cells among CD45$^+$ immune cells. (j) Percentage of CD69$^+$ activated CD8$^+$ T cells among CD45$^+$ immune cells. Data are expressed as mean +/- SD. Statistical significance was determined by ordinary one-way ANOVA. Source data are provided as a Source Data file.

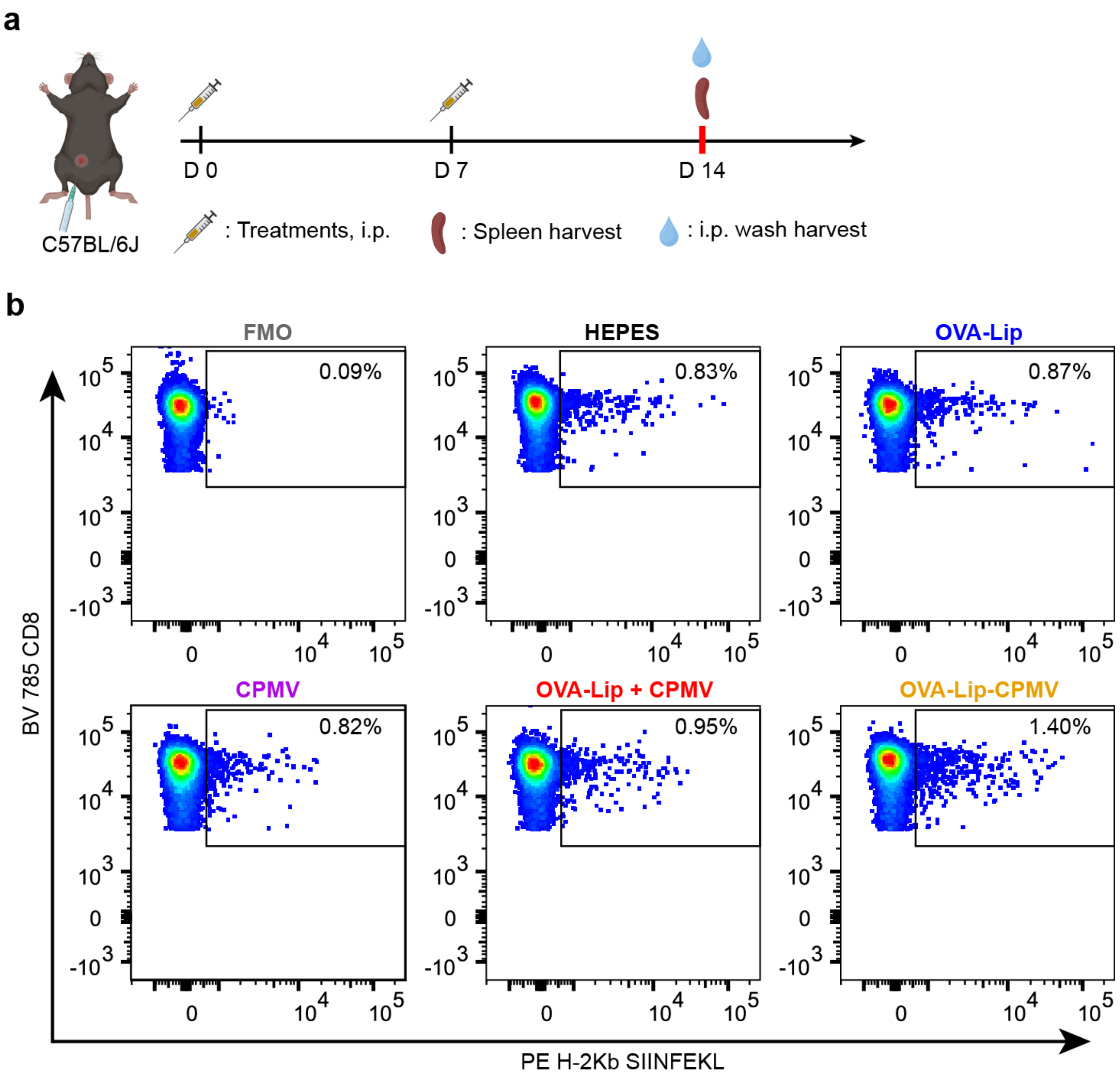

Supplementary Fig. 22. Analysis of antigen-specific immune response. (a) Schedule for OVA-Lip, CPMV, OVA-Lip + CPMV, and OVA-Lip-CPMV administration, i.p. wash and spleen collection. (b) Representative flow cytometry plots of SIINFEKL^{+} CD8^{+} T cells in the spleens of different treatment groups. A bar graph is presented in Fig. 5b. Part of (a) is generated using BioRender.

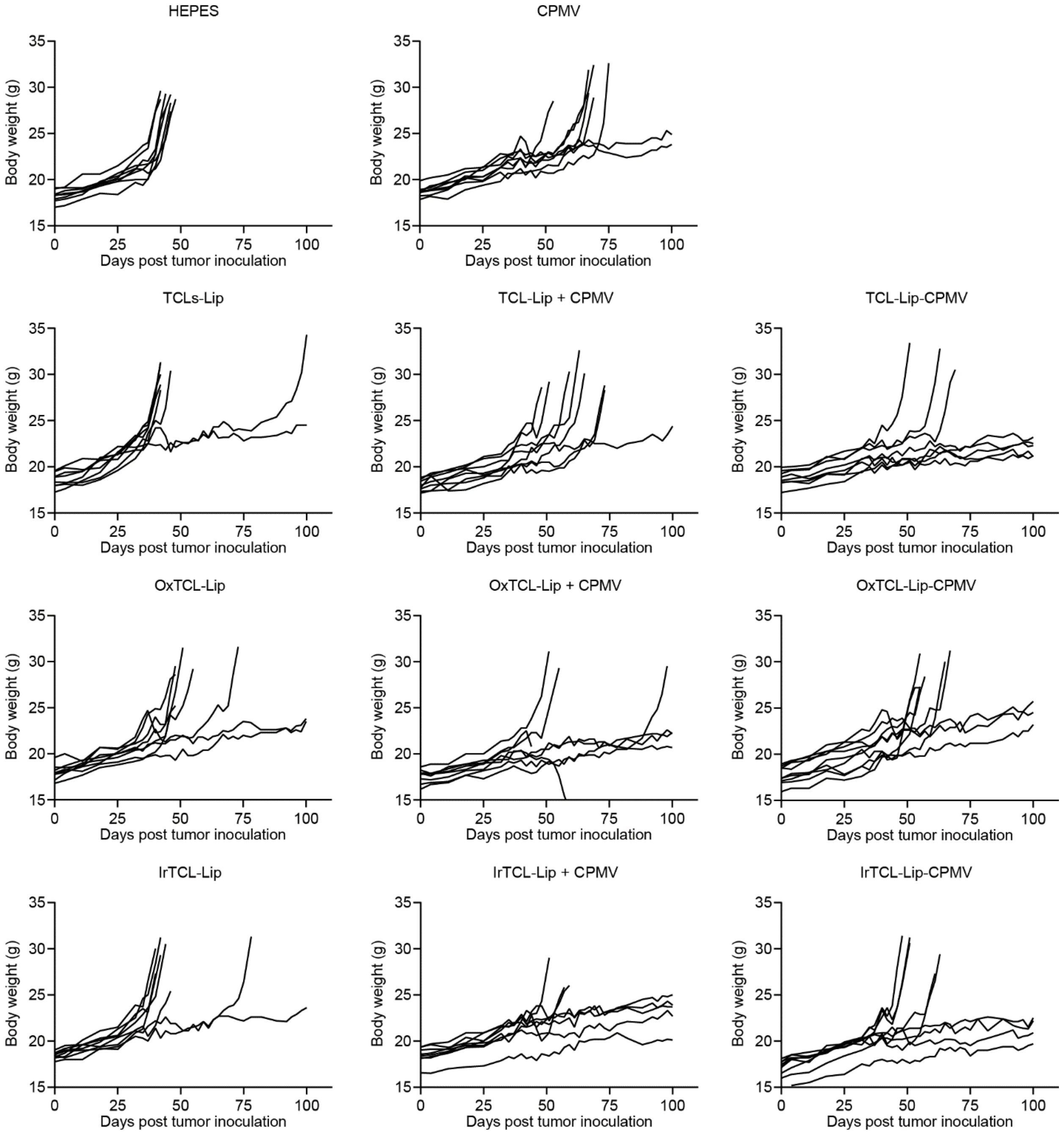

Supplementary Fig. 23. Individual body weights of all treated mice for the ovarian cancer model.

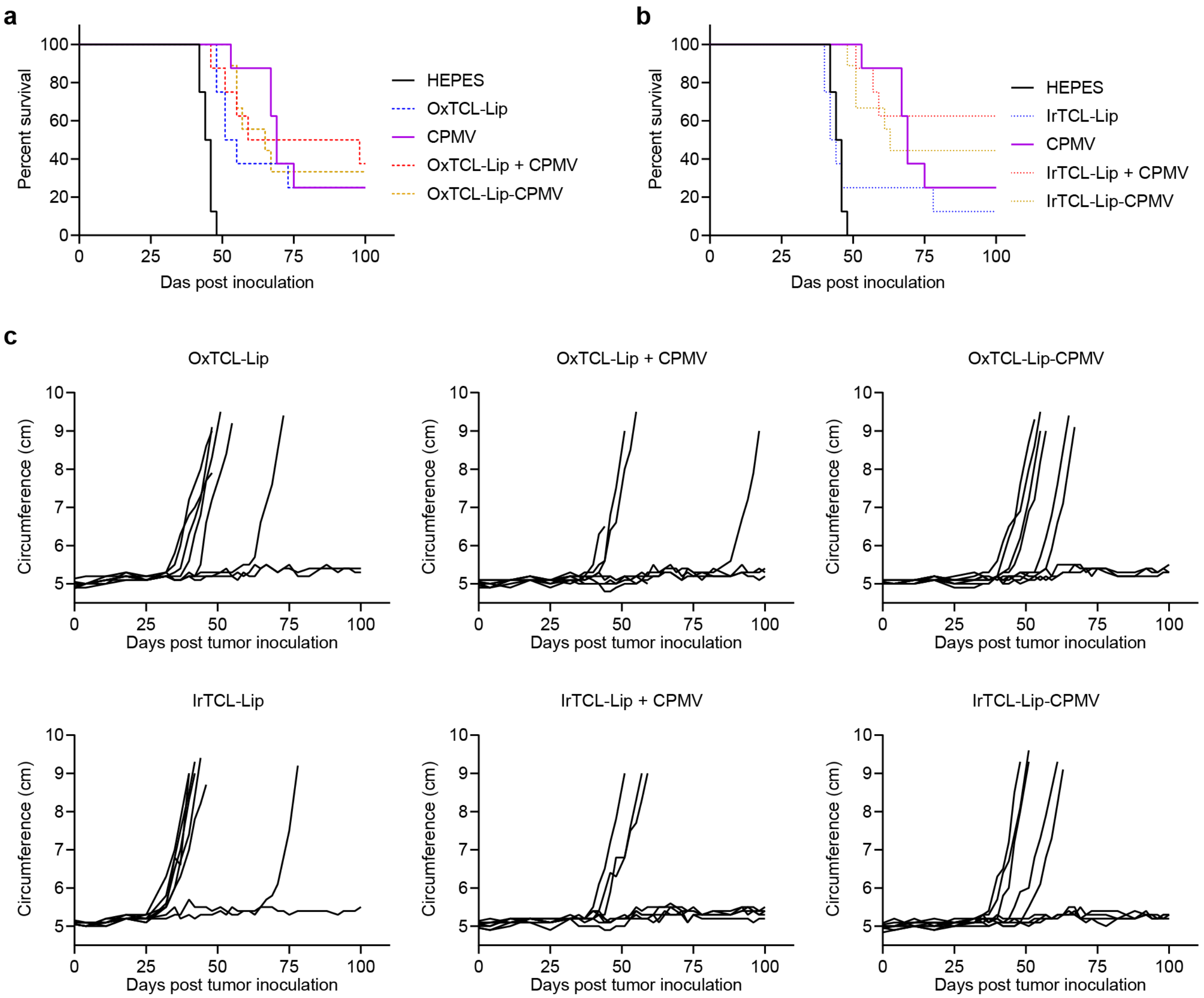

Supplementary Fig. 24. Survival rates and individual circumferences of mice treaded with oxidized and irradiated TCL. (a) Survival rates for HEPES, OxTCL-Lip, CPMV, OxTCL-Lip + CPMV, and OxTCL-Lip-CPMV groups. (b) Survival rates for HEPES, IrTCL-Lip, CPMV, IrTCL-Lip + CPMV, and IrTCL-Lip-CPMV groups. (c) Individual circumferences for mice treated with OxTCL-Lip, OxTCL-Lip + CPMV, OxTCL-Lip-CPMV, IrTCL-Lip, IrTCL-Lip + CPMV, and IrTCL-Lip-CPMV.

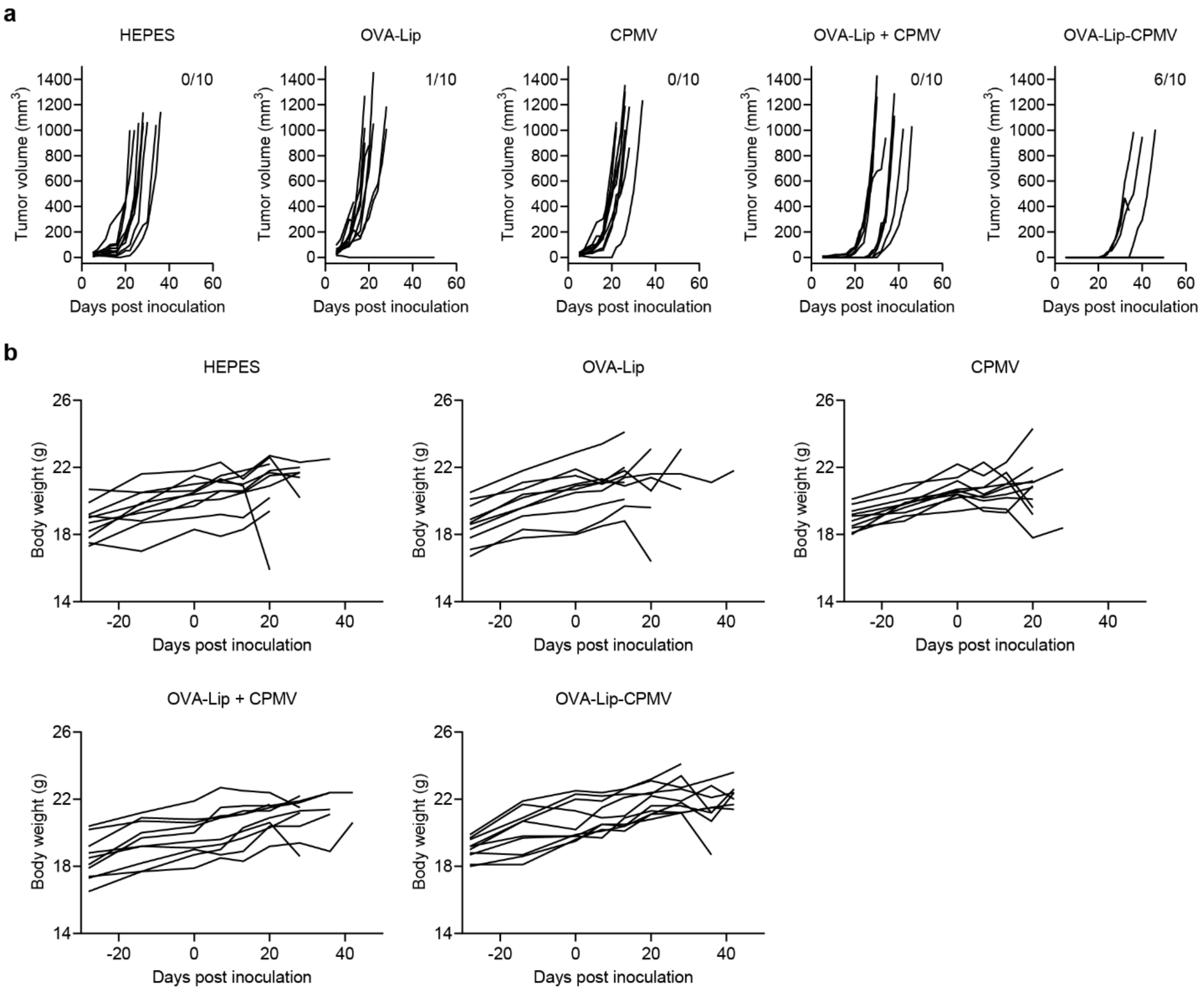

Supplementary Fig. 25. Individual tumor volumes (a) and body weights (b) for the i.d. B16F10-OVA model vaccination study in Fig. 6d.

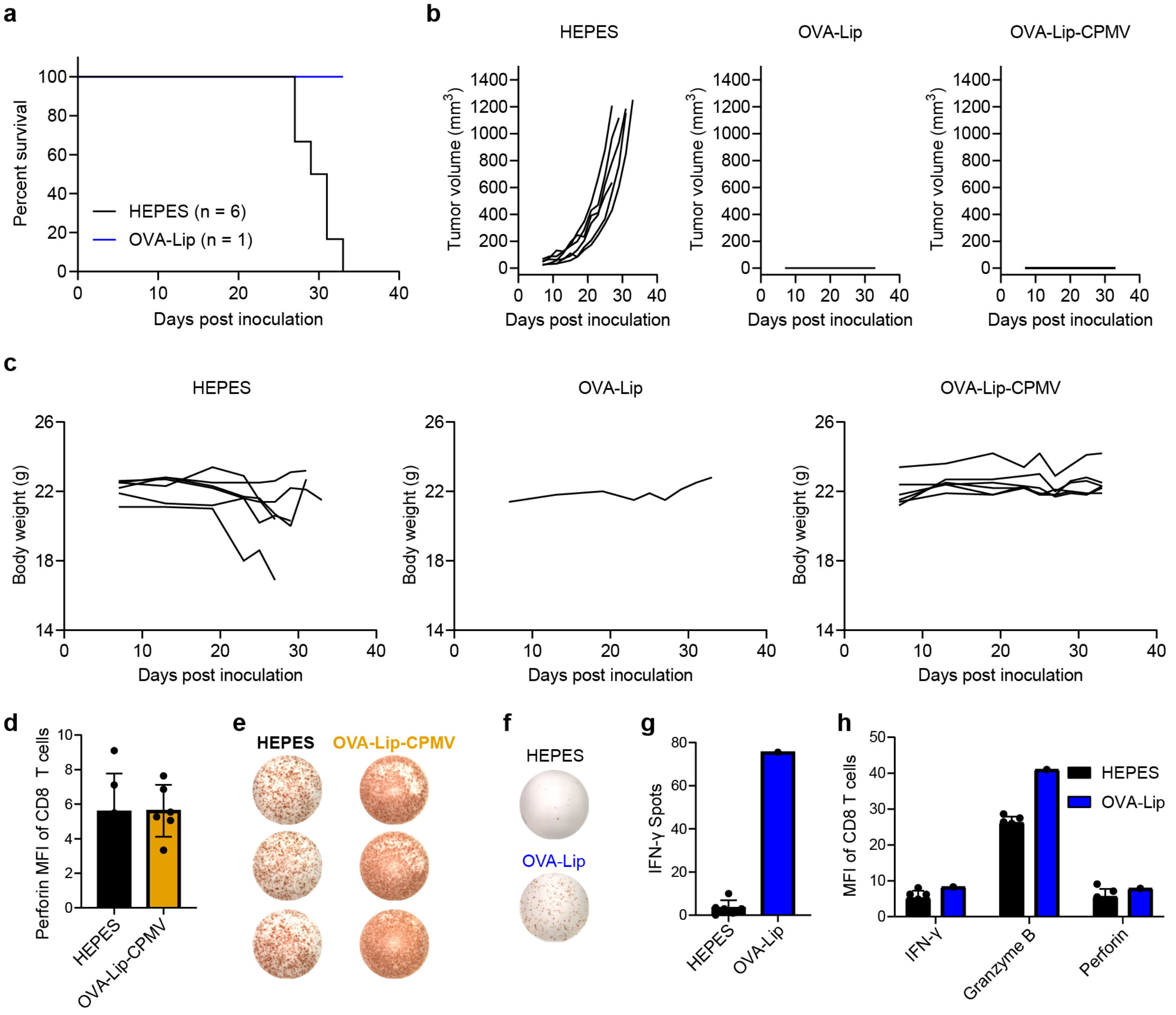

Supplementary Fig. 26. (a) Surviving animals were subjected to rechallenge; data show the survival rates for the B16F10-OVA rechallenge study (n = 6 for the Control group and n = 1 for OVA-Lip group). Individual tumor volumes (b) and body weights (c) for the B16F10-OVA rechallenge study in Fig. 6f. (d) Flow cytometry analysis of the intracellular Perforin within CD8 T cells among the splenocytes post B16F10-OVA stimulation; n = 6 independent experiments, data are expressed as mean +/- SD. (e) Representative images of an IFN-γ ELISpot assay using splenocytes from Control and OVA-Lip-CPMV groups stimulated against B16F10-OVA cells. (f) Representative images of the IFN-γ ELISpot assay for the splenocytes stimulated by SIINFEKL peptides for the Control and OVA-Lip groups. (g) Quantification of the IFN-γ spots for the Control and OVA-Lip groups, data are expressed as mean +/- SD. (h) Flow cytometry analysis of the intracellular IFN-γ, Granzyme B, and Perforin within CD8 T cells among splenocytes post B16F10-OVA stimulation, data are expressed as mean +/- SD. In the flow cytometry and ELISpot studies, only surviving mice from the B16F10-OVA challenge and rechallenge experiments (Fig. 6d-f) and control group were analyzed: OVA-Lip-CPMV (n = 6), OVA-Lip (n = 1), and Control (n =6). Source data are provided as a Source Data file (d,g,h).

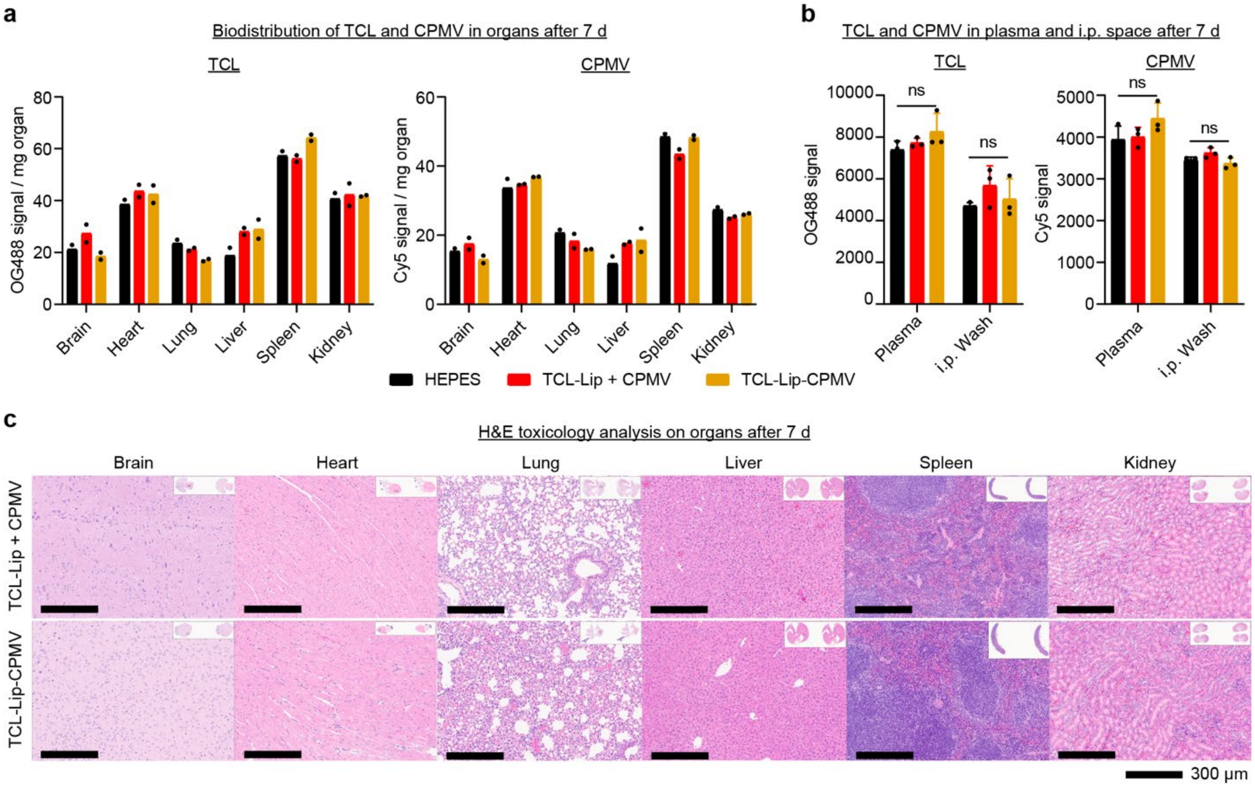

Supplementary Fig. 27. Biosafety of vaccine candidate TCL-Lip + CPMV and TCL-Lip-CPMV in healthy mice. Biodistribution of i.p. injected vaccine formulations labeled with fluorophores in various organs (a) and plasma and i.p. space (b) 7 days post injection. n = 2 independent experiments for (a) and n = 3 independent experiments for (b); data are expressed as mean +/- SD for (b). (c) H&E toxicology analysis of major organs 7 days post vaccination. Statistical significance was determined by ordinary one-way ANOVA. Source data are provided as a Source Data file (a,b).

Supplementary references

1. Prokopowicz, Z. M.; Arce, F.; Biedron, R.; Chiang, C. L.-L.; Ciszek, M.; Katz, D. R.; Nowakowska, M.; Zapotoczny, S.; Marcinkiewicz, J.; Chain, B. M., Hypochlorous acid: a natural adjuvant that facilitates antigen processing, cross-priming, and the induction of adaptive immunity. *The journal of immunology* **2010,** *184* (2), 824-835.
