# Supplementary material for: A cowpea mosaic virus adjuvant conjugated to liposomes loaded with tumor cell lysates as an ovarian cancer vaccine
Source: Nat Commun. 2025 May 30;16:5047. doi: 10.1038/s41467-025-60239-w (PMC12125389; doi:10.1038/s41467-025-60239-w)
Supplement: Supplementary file 2 — Reporting Summary [file 41467_2025_60239_MOESM2_ESM.pdf]

nature portfolio

Corresponding author(s): Nicole F. Steinmetz

Last updated by author(s): May 15, 2025

Reporting Summary

Nature Portfolio wishes to improve the reproducibility of the work that we publish. This form provides structure for consistency and transparency in reporting. For further information on Nature Portfolio policies, see our Editorial Policies and the Editorial Policy Checklist.

Statistics

For all statistical analyses, confirm that the following items are present in the figure legend, table legend, main text, or Methods section.

n/a	Confirmed	
☐	☒	The exact sample size (*n*) for each experimental group/condition, given as a discrete number and unit of measurement
☐	☒	A statement on whether measurements were taken from distinct samples or whether the same sample was measured repeatedly
☐	☒	The statistical test(s) used AND whether they are one- or two-sided<br>*Only common tests should be described solely by name; describe more complex techniques in the Methods section.*
☒	☐	A description of all covariates tested
☒	☐	A description of any assumptions or corrections, such as tests of normality and adjustment for multiple comparisons
☐	☒	A full description of the statistical parameters including central tendency (e.g. means) or other basic estimates (e.g. regression coefficient) AND variation (e.g. standard deviation) or associated estimates of uncertainty (e.g. confidence intervals)
☐	☒	For null hypothesis testing, the test statistic (e.g. *F*, *t*, *r*) with confidence intervals, effect sizes, degrees of freedom and *P* value noted<br>*Give P values as exact values whenever suitable.*
☒	☐	For Bayesian analysis, information on the choice of priors and Markov chain Monte Carlo settings
☒	☐	For hierarchical and complex designs, identification of the appropriate level for tests and full reporting of outcomes
☒	☐	Estimates of effect sizes (e.g. Cohen's *d*, Pearson's *r*), indicating how they were calculated

Our web collection on statistics for biologists contains articles on many of the points above.

Software and code

Policy information about availability of computer code

Data collection	NanoDrop 2000 spectrophotometer (Thermo Fisher Scientific)<br>ProteinSimple FluorChem R imager<br>Zetasizer Nano ZSP/Zen5600 instrument (Malvern Panalytical)<br>Talos TEM (Thermo Fisher Scientific)<br>AKTA purifier system (GE Healthcare)<br>Talos Arctica microscope (Thermo Fisher Scientific)<br>Tecan Infinite M200 plate reader<br>Xenogen IVIS 200 imaging system<br>Nikon A1R confocal microscope<br>BD Accuri C6 Plus flow cytometer<br>BD FACSCelestaTM Cell Analyzer (BD Biosciences)
Data analysis	UCSF Chimera 1.16<br>ChemDraw 20.0.0<br>NIS-Elements AR Analysis v5.30 (Nikon)<br>Immunospot S6 Entry analyzer (Cellular Technology Limited).<br>Aperio ImageScope 12.4.6<br>GraphPad Prism 10.3.1<br>FlowJo 10.7.1.

All softwares used for analysis were described in manuscript.

For manuscripts utilizing custom algorithms or software that are central to the research but not yet described in published literature, software must be made available to editors and reviewers. We strongly encourage code deposition in a community repository (e.g. GitHub). See the Nature Portfolio guidelines for submitting code & software for further information.

Data

Policy information about availability of data

All manuscripts must include a data availability statement. This statement should provide the following information, where applicable:

- Accession codes, unique identifiers, or web links for publicly available datasets
- A description of any restrictions on data availability
- For clinical datasets or third party data, please ensure that the statement adheres to our policy

All data generated in this study are available within the Article, Supplementary Information, and Source Data file. Raw data are available from the corresponding author upon request. Source Data are provided with this paper.

Research involving human participants, their data, or biological material

Policy information about studies with human participants or human data. See also policy information about sex, gender (identity/presentation), and sexual orientation and race, ethnicity and racism.

Reporting on sex and gender	N/A
Reporting on race, ethnicity, or other socially relevant groupings	N/A
Population characteristics	N/A
Recruitment	N/A
Ethics oversight	N/A

Note that full information on the approval of the study protocol must also be provided in the manuscript.

Field-specific reporting

Please select the one below that is the best fit for your research. If you are not sure, read the appropriate sections before making your selection.

- [x] Life sciences
- [ ] Behavioural & social sciences
- [ ] Ecological, evolutionary & environmental sciences

For a reference copy of the document with all sections, see nature.com/documents/nr-reporting-summary-flat.pdf

Life sciences study design

All studies must disclose on these points even when the disclosure is negative.

Sample size	No statistical methods were used to predetermine sample size. The sample sizes used in this study was based on our previous published protocols, which could generate statistic significant. (Journal of Materials Chemistry B 11.24 (2023): 5429-5441; Biomater Sci. 2020 Sep 30; 8 (19):5489-5503) Different experiments have different sample sizes. Each sample size is indicated in figure legends and methods.
Data exclusions	No data were excluded from the analysis.
Replication	Each replication size is indicated in figure legends.
Randomization	All in vivo mice experiments were randomly allocated into different experimental groups. No randomization was performed for in vitro cell culture experiments.
Blinding	Blinding was not performed and was not possible during data collection and analysis because all treatments for in vivo mice studies and in vitro cell culture studies were known and labeled on cages and plates.

Reporting for specific materials, systems and methods

We require information from authors about some types of materials, experimental systems and methods used in many studies. Here, indicate whether each material, system or method listed is relevant to your study. If you are not sure if a list item applies to your research, read the appropriate section before selecting a response.

Materials & experimental systems

n/a	Involved in the study
	☒ Antibodies
	☒ Eukaryotic cell lines
☒	Palaeontology and archaeology
	☒ Animals and other organisms
☒	Clinical data
☒	Dual use research of concern
	☒ Plants

Methods

n/a	Involved in the study
☒	ChIP-seq
	☒ Flow cytometry
☒	MRI-based neuroimaging

Antibodies

Antibodies used	anti-CD16/32 Fc block (1:500, Biolegend, 101302), PE-conjugated anti-CD11c antibody (1:100, Biolegend, 117308), Armenian hamster anti-mouse CD11c monoclonal antibody (1:100, Biolegend, 117302), rat anti-mouse F4/80 monoclonal antibody (1:100, Biolegend, 123110), rat anti-mouse B220 monoclonal antibody (1:100, Biolegend, 103202), rat anti-mouse CD3 monoclonal antibody (1:100, Biolegend, 100202), goat anti-Armenian hamster TRITC polyclonal antibody (1:500, Abcam, ab5741), goat anti-rat Alexa Fluor 555 polyclonal antibody (1:500, Invitrogen, A-21434), Pacific Blue anti-CD45 antibody (1:100, Biolegend, 103126), Super Bright 780 anti-CD11c antibody (1:100, Thermo Fisher Scientific, 78-0114-82), APC MHC-II antibody (1:100, Thermo Fisher Scientific, 17-5320-82), FITC anti-CD40 antibody (1:100, Thermo Fisher Scientific, 11-0402-82), Brilliant Violet 605 anti-CD80 antibody (1:100, Biolegend, 104729), PerCP-Cy5.5 anti-CD86 antibody (1:100, Biolegend, 105028), APC anti-CD11c antibody (1:100, Thermo Fisher Scientific, 17-0114-82), PE anti-mouse H-2Kb bound to SIINFEKL antibody (1:100, Biolegend, 141604), APC-Cy7 anti-CD3 antibody (1:100, Biolegend, 100222), FITC anti-CD4 antibody (1:100, Biolegend, 100406), Brilliant Violet 785 anti-CD8 antibody (1:100, Biolegend, 100750), Brilliant Violet 605 anti-CD44 antibody (1:100, Biolegend, 103047), APC anti-CD62L antibody (1:100, Biolegend, 104412), PerCp-Cy5.5 anti-CD69 antibody (1:100, Biolegend, 104522), PE-conjugated H-2 Kb SIINFEKL Dextramer (1:100, Immudex, JD02163), PerCp-Cy5.5 anti-IFN-γ antibody (1:100, Invitrogen, 45-7311-82), APC anti-Granzyme B antibody (1:500, Invitrogen, 17-8898-82), and PE anti-Perforin antibody (1:100, Invitrogen, 12-9392-82).<br>For all experiments using antibodies, after dilution as mentioned above for each antibody, 100 μL of the diluted antibodies were used per sample in experiments.
Validation	All antibodies used in this study are commercially available and have been extensively used in other published studies.<br>All antibodies were validated by providers and other published studies on their websites (Abcam, Biolegend, Thermo Fisher Scientific, Immudex).

Eukaryotic cell lines

Policy information about cell lines and Sex and Gender in Research

Cell line source(s)	ID8-Defb29/Vegf-a-Luc was engineered to express luciferase by previous lab members based on the ID8-Defb29/Vegf-a cell line (ACS omega 3.4 (2018): 3702-3707). ID8-Defb29/Vegf-a was a gift from Prof. Steven Fiering lab at Dartmonth College; this cell line was engineered and reported in Nature medicine 10.9 (2004): 950-958.<br>B16F10-OVA was a gift from Prof. Liangfang Zhang's Lab at UCSD. This cell line was engineered to express OVA based on B16F10 (ATCC, CRL-6475) and reported in Advanced Materials 32.30 (2020): 2001808.
Authentication	No authentication was performed in this current study.<br>The expression of luciferase was conformed VIS Spectrum BLI (PerkinElmer) imaging (ACS omega 3.4 (2018): 3702-3707).<br>The expression of ovalbumin in B16F10-OVA cells was confirmed by westblot and flow cytometry in the published article (Advanced Materials 32.30 (2020): 2001808).
Mycoplasma contamination	All cell lines were healthy and no contamination was observed during the cell culture.
Commonly misidentified lines (See ICLAC register)	No commonly misidentified lines were used in this study.

Animals and other research organisms

Policy information about studies involving animals; ARRIVE guidelines recommended for reporting animal research, and Sex and Gender in Research

Laboratory animals	7-week-old female C57BL/6J mice from Jackson Laboratories. All mice were housed in a light-controlled room with a 12h light/12h dark cycle at 18-23 °C and 50% ± 10% humidity, and all mice had free access to water and diet.
Wild animals	No wild animals.
Reporting on sex	Only female mice were studied because this study focused on ovarian cancer, which only afflict women.
Field-collected samples	N/A

Ethics oversight	All studies involving mice were carried out in accordance with the guidelines of the Institutional Animal Care and Use Committee (IACUC) of the University of California, San Diego (UCSD) under the protocol number S18021 and were approved by the Animal Ethics Committee of UCSD.

Note that full information on the approval of the study protocol must also be provided in the manuscript.

Dual use research of concern

Policy information about dual use research of concern

Hazards

Could the accidental, deliberate or reckless misuse of agents or technologies generated in the work, or the application of information presented in the manuscript, pose a threat to:

No	Yes	
☒	☐	Public health
☒	☐	National security
☒	☐	Crops and/or livestock
☒	☐	Ecosystems
☒	☐	Any other significant area

Experiments of concern

Does the work involve any of these experiments of concern:

No	Yes	
☒	☐	Demonstrate how to render a vaccine ineffective
☒	☐	Confer resistance to therapeutically useful antibiotics or antiviral agents
☒	☐	Enhance the virulence of a pathogen or render a nonpathogen virulent
☒	☐	Increase transmissibility of a pathogen
☒	☐	Alter the host range of a pathogen
☒	☐	Enable evasion of diagnostic/detection modalities
☒	☐	Enable the weaponization of a biological agent or toxin
☒	☐	Any other potentially harmful combination of experiments and agents

Plants

Seed stocks	Cowpea seeds/plants No.5 (Vigna unguiculata)
Novel plant genotypes	N/A
Authentication	N/A

Flow Cytometry

Plots

Confirm that:

☒ The axis labels state the marker and fluorochrome used (e.g. CD4-FITC).

☒ The axis scales are clearly visible. Include numbers along axes only for bottom left plot of group (a 'group' is an analysis of identical markers).

☒ All plots are contour plots with outliers or pseudocolor plots.

☒ A numerical value for number of cells or percentage (with statistics) is provided.

Methodology

Sample preparation	Detailed in materials and methods
Instrument	BD Accuri C6 Plus flow cytometer (BD Biosciences), BD FACSCelestaTM Cell Analyzer (BD Biosciences).
Software	Flowjo_v10.7
Cell population abundance	>=1 million cells were prepared in each sample
Gating strategy	Presented in the supplementary information

☒ Tick this box to confirm that a figure exemplifying the gating strategy is provided in the Supplementary Information.
